# Fabrication Strategies for Bioceramic Scaffolds in Bone Tissue Engineering with Generative Design Applications

**DOI:** 10.3390/biomimetics9070409

**Published:** 2024-07-05

**Authors:** Bilal Cinici, Sule Yaba, Mustafa Kurt, Huseyin C. Yalcin, Liviu Duta, Oguzhan Gunduz

**Affiliations:** 1 Department of Mechanical Engineering, Faculty of Technology, Marmara University, Istanbul 34890, Turkey; bilal@ayeminnovation.com (B.C.); m.kurt@marmara.edu.tr (M.K.); 2 Center for Nanotechnology & Biomaterials Application and Research (NBUAM), Marmara University, Istanbul 34890, Turkey; 3 AYEM Innovation Anonim Sirketi, Cube Incubation Center, Technopark Istanbul, Istanbul 34890, Turkey; suleeyaba@gmail.com; 4 Biomedical Research Center, Qatar University, Doha 2713, Qatar; hyalcin@qu.edu.qa; 5 Department of Biomedical Science, College of Health Sciences, QU Health, Qatar University, Doha 2713, Qatar; 6 Department of Mechanical and Industrial Engineering, Qatar University, Doha 2713, Qatar; 7 Lasers Department, National Institute for Lasers, Plasma and Radiation Physics, 077125 Magurele, Romania; 8 Department of Metallurgical and Materials Engineering, Faculty of Technology, Marmara University, Istanbul 34890, Turkey

**Keywords:** bone tissue engineering, 3D scaffold, bioceramics, composites, generative design

## Abstract

The aim of this study is to provide an overview of the current state-of-the-art in the fabrication of bioceramic scaffolds for bone tissue engineering, with an emphasis on the use of three-dimensional (3D) technologies coupled with generative design principles. The field of modern medicine has witnessed remarkable advancements and continuous innovation in recent decades, driven by a relentless desire to improve patient outcomes and quality of life. Central to this progress is the field of tissue engineering, which holds immense promise for regenerative medicine applications. Scaffolds are integral to tissue engineering and serve as 3D frameworks that support cell attachment, proliferation, and differentiation. A wide array of materials has been explored for the fabrication of scaffolds, including bioceramics (i.e., hydroxyapatite, beta-tricalcium phosphate, bioglasses) and bioceramic–polymer composites, each offering unique properties and functionalities tailored to specific applications. Several fabrication methods, such as thermal-induced phase separation, electrospinning, freeze-drying, gas foaming, particle leaching/solvent casting, fused deposition modeling, 3D printing, stereolithography and selective laser sintering, will be introduced and thoroughly analyzed and discussed from the point of view of their unique characteristics, which have proven invaluable for obtaining bioceramic scaffolds. Moreover, by highlighting the important role of generative design in scaffold optimization, this review seeks to pave the way for the development of innovative strategies and personalized solutions to address significant gaps in the current literature, mainly related to complex bone defects in bone tissue engineering.

## 1. Introduction

In modern medicine, the quest to repair and regenerate tissues and organs has led to groundbreaking advancements in regenerative medicine. Central to this progress is the field of tissue engineering. Among various tissues, bone represents a dynamic, highly vascularized tissue that plays a crucial role in providing structural support and facilitating movement while protecting vital organs. Besides these critical functions and structural complexity [1], bone has an intrinsic ability to heal and remodel. However, significant defects due to trauma, disease, or surgical intervention often exceed bone’s natural regenerative capacity. Traditional medical interventions (i.e., autografts, allografts, and xenografts) to restore its function and integrity are therefore necessary [2,3]. Autografts, considered the gold standard, offer superior biocompatibility and osteogenic potential but are limited by donor site morbidity and supply constraints. On the other hand, allografts, and xenografts, though more readily available, carry the risks of immune rejection and disease transmission [4]. In this respect, the development of effective bone regenerative materials is essential to address the limitations of traditional treatments and improve patient outcomes.

Within the context of tissue engineering, the terms “scaffold” or “bio-scaffold” refer to three-dimensional (3D) structures specifically designed to elicit biological responses and support cell attachment, proliferation, and differentiation. In this context, the regeneration or formation of new tissue is thereby facilitated. These scaffolds, which can be made from either natural or synthetic materials, are designed to mimic the extracellular matrix, and they offer mechanical support and facilitate the exchange of nutrients and waste [5].

Thus, a wide array of materials has been explored for the fabrication of bio-scaffolds, each offering unique properties and functionalities tailored to specific applications. Thus, bio-scaffolds made of biocompatible materials such as polymers, ceramics, metals, and composites meet the requirements for successful tissue regeneration. Among these materials, mineral fillers such as bioceramics (i.e., hydroxyapatite—HA, beta-tricalcium phosphate—β-TCP) have emerged as particularly promising candidates used in scaffold fabrication for bone tissue engineering. This is due to their excellent biocompatibility, bioactivity, and similarity to the mineral component of natural bone [6,7,8]. Bioceramics are a class of ceramic materials specifically designed for medical and dental applications. They can interact with biological tissue without eliciting an adverse response and have the ability to support bone in-growth and regeneration [9]. HA, closely resembling the mineral component of bone, was demonstrated to promote cell adhesion and proliferation [10]. However, its low degradation rate can limit the natural remodeling process of bone. On the other hand, β-TCP exhibits a higher resorption rate, which can match the natural bone regeneration process more closely. However, its rapid degradation rate may compromise the scaffold’s mechanical integrity before sufficient new tissue is formed [11]. It is important to mention that these bio-scaffolds must meet stringent standards for biocompatibility, porosity, and mechanical strength. Therefore, a scaffold’s mechanical integrity is critical, allowing it to survive surgical manipulation during implantation and guaranteeing complete remodeling at the implantation site. To assist cell adhesion and development, ideal scaffolds have precise compressive and tensile strengths, as well as optimum porosity and pore size. Furthermore, these scaffolds transport cells, growth factors, genes, antibodies, medicines, and nanoparticles. This allows for targeted therapy at the injection site. Surface protein, mineral, and biomolecule functionalization improves cell adhesion and proliferation.

Bioactive mineral fillers, such as bioglasses (BGs) and calcium silicate (CaSi) materials, are extensively used in scaffold fabrication for tissue engineering due to their superior bioactivity and osteoconductivity. BGs are renowned for their ability to bond with bone and soft tissues. They enhance cellular responses and promote rapid bone regeneration [12]. Additionally, BGs can stimulate angiogenesis, which is crucial for tissue repair. However, their brittleness and relatively slow degradation rate can limit their use in dynamic load-bearing applications [13]. Similarly, CaSi materials exhibit excellent bioactivity and promote bone cell proliferation and differentiation [14]. In physiological conditions, they can form HA on their surfaces, which facilitates bone integration [15]. Despite these benefits, the high degradation rate of CaSi can lead to a premature loss of structural integrity, and their brittleness remains a challenge.

Polymers and biopolymers are pivotal in scaffold fabrication for tissue engineering due to their functional versatility and tunable properties. Synthetic polymers like polylactic acid (PLA), polyglycolic acid (PGA), and polycaprolactone (PCL) are widely used due to their mechanical strength, customizability, and ease of processing. However, their hydrophobic nature can impede cell attachment and their degradation can lead to acidic by-products, which can potentially cause inflammation [16]. In contrast, natural biopolymers such as collagen and chitosan provide excellent biocompatibility and support for cell adhesion and proliferation. Thus, they can closely mimic the natural extracellular matrix. Yet, these materials often lack the necessary mechanical strength for load-bearing applications and can exhibit variability in their properties [17]. Hence, the choice between synthetic and natural polymers often involves a trade-off between mechanical properties and biological performance.

In recent years, there has been growing interest in the development of composite materials that combine bioceramics’ properties with those pertaining to polymers to enhance the scaffold’s mechanical properties and functionality [18,19,20]. Notably, HA–polymer or BGs–polymer composites have gained traction as a versatile class of biomaterials used to fabricate bio-scaffolds with tailored mechanical properties and enhanced bioactivity. One should mention here that the term “bioactivity”, in the context of scaffold fabrication, refers to the ability of a material to induce a biological response that leads to the formation of a bond between the material and the surrounding tissue. These composites offer a synergistic combination of the bioactivity of HA and the flexibility and processability of polymers. This results in scaffolds that can promote cell adhesion, proliferation, and differentiation. Moreover, HA–polymer composites exhibit improved mechanical strength and handling characteristics compared to pure bioceramics. These enhanced properties make them well-suited for various bone tissue-engineering applications [21,22,23].

Several fabrication methods have been employed to create bioceramic scaffolds, ranging from conventional techniques to advanced additive-manufacturing approaches. Conventional methods such as solvent casting [24], particulate leaching [25], and freeze-drying [26] enable the fabrication of porous scaffolds with controlled porosity and pore size distribution. These techniques offer simplicity and scalability, which makes them suitable for producing bulk scaffolds for bone tissue engineering. In contrast, additive manufacturing (AM), including 3D printing and electrospinning, is a cutting-edge process that involves layer-by-layer material deposition [27]. Subtractive manufacturing, on the other hand, includes sculpting a solid mass by cutting, drilling, or milling [28]. Both additive- and subtractive-manufacturing techniques provide precise control over the scaffold architecture and composition. This allows for the fabrication of complex, patient-specific scaffolds with tailored mechanical properties and bioactivity [29,30]. One notes that techniques like selective laser sintering, material jetting, and/or fused deposition modeling actively contribute to the development of bio-scaffolds using user-defined computer-aided design (CAD) models [31,32]. Medical imaging tools such as Computer Tomography and Magnetic Resonance Imaging are being used to customize structures for individual patient needs [33,34].

Generative design represents a paradigm shift in the field of scaffold design and fabrication. It offers a novel approach to optimize scaffold architectures for specific applications [35,36]. Rooted in computational algorithms and iterative optimization techniques, generative design enables the exploration of vast design spaces to identify optimal scaffold configurations that maximize mechanical strength, biological performance, and tissue integration. By harnessing the power of generative design, researchers can design and fabricate highly customized scaffolds with enhanced functionality and regenerative potential. The field of bone tissue engineering is therefore advancing toward personalized medicine and improved clinical outcomes.

The aim of this study is to provide an overview of the current state-of-the-art in the field of bioceramic scaffolds’ fabrication for bone tissue engineering, with an emphasis on the application of 3D-printing technologies and generative design principles. By analyzing the latest research findings and technological advances in scaffold design and fabrication, this review will thus emphasize the unique potential of bioceramic scaffolds and generative design technologies as promising approaches for bone regeneration and repair, as well as in bone shape development. Here, 3D technologies and various printing processes have proven invaluable in the examination of bone tissue production, enabling the investigation of diverse forms such as square, round, and groin structures. Moreover, by highlighting the role of generative design in scaffold optimization, this review seeks to pave the way for the development of innovative strategies and personalized solutions to address significant gaps in the current literature related to both complex bone defects and fractures in clinical practice.

## 2. Scaffolds for Bone Tissue Engineering

**Figure 1:**
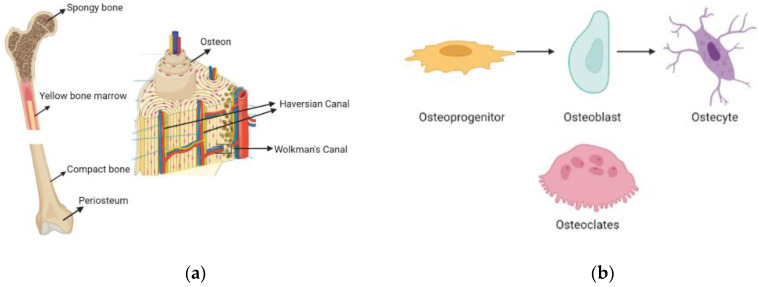
(**a**) Bone structure, and (**b**) types of cells that are found within bone tissue.

As shown in Figure 1a, the exploration of the intricate geography of bone formation reveals a complex network of interwoven nerves, arteries, marrow, and the encircling periosteum. Bone comprises a broad array of specialized components that range from macro to micro sizes, and it plays a foundational role in providing support to the body. Among these elements, bone marrow has a critical role in blood production.

Furthermore, bones exhibit exceptional storage capacity, which accounts for 99% of the body’s calcium reserves, while also playing important roles in vital physiological functions, including muscle contraction, blood clotting, and nerve signal transmission.

Bone tissues consist of a harmonious combination of inorganic carbonaceous compounds, primarily the extracellular matrix (ECM) and collagen [37], alongside organic molecules, which comprise approximately 5–10% water and 3% lipids. With 50–70% inorganic components, predominantly HA, and type I collagen accounting for 97% of the ECM by weight, bone tissue is naturally a composite material [38,39].

HA, characterized by its crystalline structure composed of calcium and phosphate, along with additional components like citrate, plays a significant role in shaping the complex mineralized structure of bone tissue. Despite its intricate mineralization process, bone remains a living structure, which houses blood vessels and a variety of cell types that are crucial for its production and regeneration (as shown in Figure 1b). Osteoprogenitors, osteoblasts, osteocytes, and osteoclasts are important cell types in this intricate process.

Osteoprogenitors, found in the periosteum, endosteum, and Haversian canals, differentiate into bone tissue when triggered by stimulation-induced cell production. Osteoblasts play a pivotal role in bone development as they are specialized in synthesizing and depositing the organic matrix of bone tissue, particularly type 1 collagen fibers. Osteocytes, which constitute 90–95% of bone cells, are vital structures that significantly contribute to maintaining bone viability, although they can degenerate over time. Derived from the fusion of monocytes in the bloodstream, osteoclasts are responsible for bone resorption and the subsequent degradation of bone tissue.

Scanning techniques provide crucial parameters to assess bone structure, including metrics such as the bone density, surface density, trabecular thickness, separation, and number, along with non-metric parameters like the structural pattern index, structural anisotropy, and joint density (which are more visually assessed [40]). Successful bone tissue-engineering applications rely on meticulous consideration of factors such as the pore size and shape, directional mechanical properties, biocompatibility, and the creation of an environment conducive to cell cultures.

**Figure 2:**
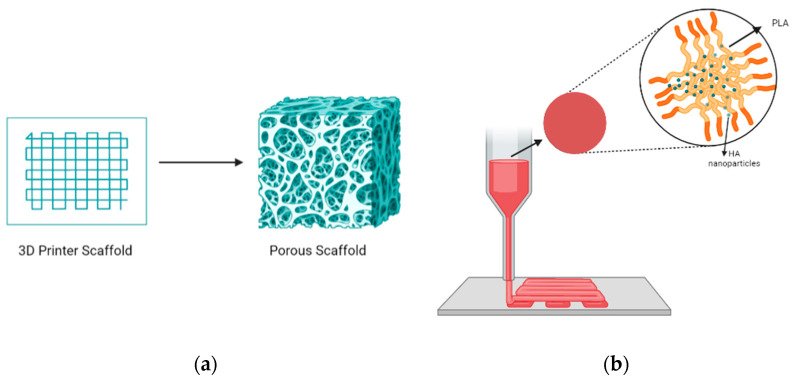
(**a**) Porous scaffold manufacturing, and (**b**) polylactic acid (PLA)/hydroxyapatite (HA) scaffold manufacturing by 3D-printing method.

Pore structures, which are among the most important factors to consider, play a vital role in the design of implantable scaffolds. These structures must be carefully designed to facilitate cell exchange and promote optimal cell proliferation and growth. As shown in Figure 2a, porous structures should have high porosity and interconnectedness. In the context of scaffold fabrication for tissue-engineering applications, the optimal pore size and porosity are crucial parameters that significantly affect cell infiltration, nutrient diffusion, and tissue growth. The optimal pore size for bone tissue engineering is generally considered to be in the range of 100–500 μm. Pores within this range facilitate cell migration, vascularization, and bone in-growth, with larger pores promoting better vascularization and smaller pores enhancing cell attachment and proliferation [41,42]. The porosity of scaffolds, which refers to the fraction of the scaffold’s volume that is void space, should ideally be between 50 and 90%. High porosity enhances nutrient and oxygen diffusion, promotes waste removal, and provides more surface area for cell attachment. However, maintaining mechanical strength at high porosity levels can be challenging. But this necessitates a balance between porosity and structural integrity [43,44]. Therefore, selecting an appropriate pore size that enhances cell interaction and promotes biocompatibility is crucial for the success of a scaffold when used in bone tissue-engineering applications [45,46].

The anisotropic nature of bone tissue, influenced by its irregular fiber arrangement, plays a central role in the design of biomedical structures. This directional variability enables bones to flexibly adapt to different loads. Understanding this aspect is vital for effective design, especially considering the irregular orientation of the fibers. Anisotropy, which arises from the varying directions of the fibers, enables dynamic responses to stress. Young’s modulus, which measures material elasticity, underscores these mechanical principles. Considering these aspects is essential to the design of porous structures that effectively mimic bone tissue, which further offers promising prospects for future biomedical applications.

The macro- and micromorphology of bone are critical parameters that guide the design of a scaffold and influence its effectiveness in supporting new tissue growth. The macromorphology involves the overall shape, size, and architecture of the scaffold, which must be designed to fit the defect site and provide sufficient mechanical support. On the other hand, micromorphology focuses on the micro-scale features such as the porosity, pore size, and surface roughness, which are crucial for cell attachment, proliferation, and differentiation. Scaffolds intended for critical size bone defects, post-extractive bone defects, and atrophic sinus bone defects must facilitate proper bone regeneration. In this respect, histological studies are of key importance and have shown that for both the mandible and maxilla, areas of mineralization are crucial for effective bone regeneration.

Critical-sized bone defects are those that cannot heal spontaneously and require intervention for regeneration. Histological studies have demonstrated that these defects often exhibit significant loss of bone matrix and mineral content. This poses a challenge for scaffold design. Therefore, these defects require scaffolds that can mimic the complex hierarchical structure of bone and provide both mechanical support and a conducive environment for bone regeneration [47].

Post-extractive bone defects occur after tooth extraction and can lead to significant alveolar bone loss if not properly managed. Histological analysis of human bone tissue around immediately loaded implants treated with a biphasic calcium phosphate [48] demonstrated rapid bone formation and minimized resorption. It was therefore indicated that the scaffold’s surface properties played a crucial role in supporting early bone healing and maintaining peri-implant bone stability.

Atrophic sinus bone defects, often seen in the posterior maxilla (where sinus pneumatization and bone resorption occur post-extraction), require scaffolds that can support significant vertical bone growth and sinus floor elevation. In Ref. [49], atrophic maxillae were shown to pose surgical and prosthetic challenges due to the horizontal and vertical bone loss. This study used specially designed CAD/CAM-manufactured allogeneic bone blocks for augmentation, which showed promising results in providing a stable base for subsequent implant placement. However, the process remains complex and requires careful planning and execution to achieve optimal outcomes.

A study published in Ref. [50] demonstrated the enhanced bone regeneration capabilities of HA-PCL composite scaffolds in a critical-sized defect (rat model). These scaffolds were 3D-printed using a Voronoi design, which allowed for improved mechanical strength and biological performance compared to individual components. The results of in vitro and in vivo testing showed that the HA-PCL scaffolds significantly improved the bone regeneration and mechanical properties. This was particularly evident when the scaffolds were combined with bone grafts, which highlighted their potential for clinical applications in bone tissue engineering. Additionally, another study published in Ref. [51] used marine plankton-derived HA in combination with PCL to create porous 3D scaffolds. These scaffolds exhibited superior cell adhesion, proliferation, and bone regeneration in a rabbit calvarial defect model compared to scaffolds made from PCL alone. This suggests that the incorporation of HA into PCL scaffolds significantly enhances their biological performance and mechanical strength.

The use of scaffolds incorporating bone morphogenetic proteins (BMPs) for enhanced bone regeneration and preservation of the alveolar ridge height was also reported [52]. It was thus demonstrated that scaffolds loaded with BMPs, particularly BMP-2, showed significant improvements in bone regeneration in critical-sized defects. The incorporation of BMPs into the scaffolds promoted rapid osteoinduction, which lead to increased bone volume and density. These are crucial to maintain the alveolar ridge height post-extraction. The findings suggested that BMP-loaded scaffolds can be a promising approach for dental applications, providing both structural support and biological cues for effective bone healing and ridge preservation.

Another study [53] evaluated the clinical and radiological outcomes of vertical bone augmentation using cortico-cancellous iliac bone grafts enriched with bone marrow aspirates. The enriched bone marrow aspirates were mixed with autogenous bone chips and deproteinized bovine bone mineral, then covered with resorbable membranes. The study reported successful vertical augmentation and the maintenance of sufficient bone volume for implant placement after six months. The obtained results highlighted the potential of this method for effective sinus lift outcomes and subsequent dental implant procedures.

The results of two human histological studies [54,55] have contributed to a better understanding of bone–implant interfaces. It is important to mention that, in both studies, the composition and mineralization of human bone were analyzed through an innovative protocol technique using environmental scanning electron microscopy connected with energy dispersive X-ray spectroscopy (ESEM-EDX). In the first study [54], the obtained results demonstrated the fast formation of compact bone tissue after seven months from the implant placement. Thus, active bone remodeling was still present after seven months. ESEM-EDX was found to be a suitable technique for obtaining more complete information on the microchemistry composition and density/mineralization of bone around implants. In terms of clinical significance, maxillary and mandibular peri-implant bone revealed different mineralization patterns, which means different healing times. The second study [55] focused on the differences in bone morphology around loaded vs. unloaded implants. Using advanced imaging techniques, this study demonstrated that loaded implants exhibited significantly higher bone density and better-organized bone trabeculae compared to unloaded implants. This suggests that mechanical loading plays a crucial role in bone remodeling and mineralization around implants. One should mention though that the limitation of this study was the use of small-diameter implants. Both studies underscored the importance of the mechanical loading and surface properties of implants in promoting bone regeneration and osseointegration. Future scaffold designs for bone tissue engineering should incorporate these findings to optimize the clinical outcomes in the treatment of various bone defects.

Modern scaffold structures are fabricated using various methods, including cutting-edge additive-manufacturing techniques such as fused deposition modeling (FDM), 3D printing, stereolithography (SLA), and selective laser sintering (SLS). Additionally, rapid fabrication methods like thermal phase separation, electrospinning, freeze-drying, gas foaming, and particle leaching/solvent deposition are also employed [56].

Utilizing innovative “generative design” techniques in scaffold manufacturing enables the creation of lightweight and durable structures. In contrast to conventional approaches, generative design, powered by advanced computer algorithms, expedites the fabrication of intricate geometries [57]. In modern production, the seamless convergence of design and manufacturing, particularly additive printing, allows for the instantaneous manufacturing of durable parts with exquisite features.

**Figure 3:**
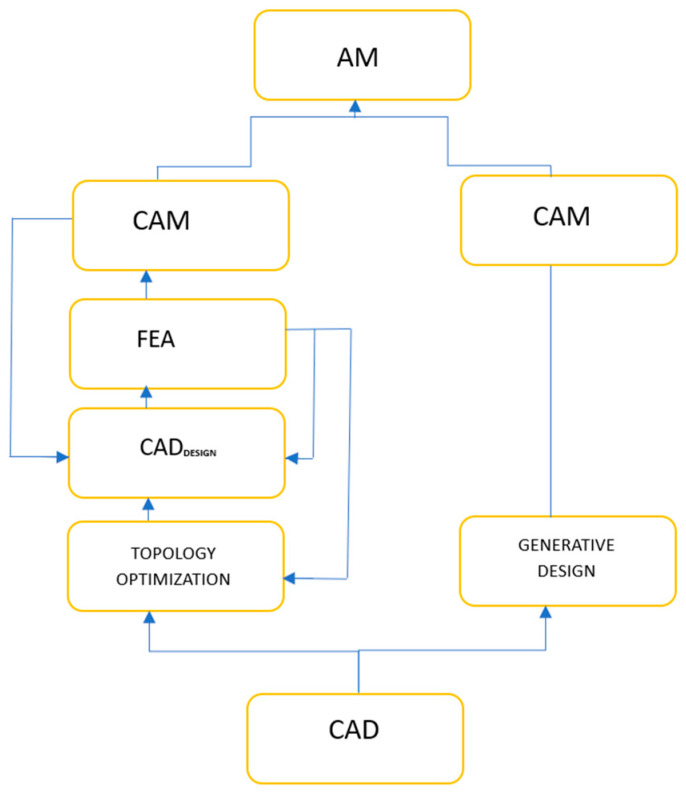
Topology optimization and generative design diagram.

Although “generative design” and topology optimization may seem similar, they operate within different contexts and utilize different program infrastructures and methodologies (Figure 3). Topology optimization starts by defining the optimization region within the computer-aided drawing model and setting boundary criteria. This approach provides effective design flexibility that can adapt as manufacturing technologies evolve [58]. Topology optimization aims to generate optimal components by preserving areas that require the highest strength while eliminating low-force-density zones. The resulting structure often includes sophisticated internal features that surpass the capabilities of older methods [59].

“Generative design” mimics natural development and begins by establishing design objectives. Designers utilize a template to define the layout of the product and consider factors such as loads, supports, materials, and manufacturing processes. Optimal solutions meeting specific limitations are developed through artificial intelligence and machine learning in an iterative process. It is important to emphasize that “generative design” does not operate independently but rather complements topology optimization to generate more data from fewer design inputs [60]. These design methodologies aim to create porous structures for applications in bone tissue engineering.

In scaffold manufacturing, the choice of materials is of paramount importance, as it dictates the ideal manufacturing methods. Although the dependence on specific materials has diminished over time, certain limitations still persist. Materials utilized in scaffolding production are typically classified into four categories: metals, ceramics, polymers, and composite materials. Furthermore, these materials are subdivided into the organic and inorganic categories based on their chemical composition. When determining the appropriate production method for scaffold structures, it is crucial to consider the classification of materials [61,62]. In bone tissue engineering, a diverse array of materials is employed, with a particular emphasis on inorganic materials and polymers. Inorganic materials, including HA, BGs, calcium phosphate (CaP), titanium dioxide, silicon dioxide, and alumina, zirconia, among others, are used to tailor the mechanical properties or biocompatibility of the targeted structures [62]. While bioceramics demonstrate effectiveness in osteoconduction, particularly under compressive loads, they may exhibit brittle behavior when subjected to tensile stresses [38].

Another category of materials is represented by polymers, which are classified into two main groups: natural and synthetic. Natural polymers are derived from animal sources, microorganisms, or plants. These materials, whose chemical structures can be customized, possess the notable characteristic of not generating cell-damaging by-products during enzymatic degradation. This advantageous attribute makes them particularly attractive for applications in bone tissue engineering [63]. However, the rate of degradation of these materials is difficult to control, and their mechanical strength should be increased [64]. Alginate, collagen, cellulose, chitosan, fibrin, and gelatine are just a few examples of natural polymers.

Among synthetic polymers, notable examples include PLA, PCL, and poly-lactic-co-glycolic acid (PLGA). The biodegradability and biocompatibility of these materials must undergo careful evaluation [38]. Composite materials are extensively used to fulfil various requirements and their application scope being broadened with organic–inorganic composites is particularly prevalent in scaffolding structures. In such composites, the polymer component provides flexibility, while the inorganic component enhances the stiffness and durability [65].

Among these materials, bioceramics have been the focus of intensive research to produce artificial bone tissues. Therefore, in the next section, the investigation of the characteristics of these biomaterials should both (i) elucidate their pivotal role in scaffold fabrication and (ii) offer valuable insights into the advancements in bone tissue engineering. Thus, a comprehensive understanding of the field and guidance for future research endeavors geared toward more effective therapeutic strategies able to enhance patient outcomes are provided.

## 3. Bioceramics Used in Bone Tissue Design

In recent years, ceramic materials have become increasingly prominent in the biomedical field for both skeletal repair and reconstruction purposes. These ceramics, specifically tailored for medical applications, are referred to as bioceramics. Bioceramics have gained popularity in bone tissue research owing to their easy preparation, favorable biodegradability, and osteogenic bioactivity, which stimulates bone formation. Moreover, their remarkable chemical and mechanical properties, including improved osteoconductive behavior, wear resistance, and biocompatibility, render them valuable for bone restoration endeavors.

Bioceramics alone may not yield optimal outcomes for 3D-printed bone scaffolds. Consequently, polymers are generally preferred as the organic component of bone tissue engineering, with bioceramics serving as the inorganic component. Among the bioceramics frequently utilized for this purpose are HA, β-TCP, BGs, and calcium silicate (CaSi). In the following paragraphs, some characteristics of these biomaterials will be briefly introduced.

### 3.1. Hydroxyapatite

HA, a bioceramic material with the complex chemical formula Ca_10_(PO_4_)_6_(OH)_2_, can be synthesized through synthetic routes or extracted as a powder from natural sources [66,67,68,69,70] like fish waste [71], egg shells, and seashells [72,73]. Each technique used to produce pure stoichiometric synthetic HA requires careful control of processing parameters such as the pH (the pH level significantly influences the synthesis process and affects the phase purity and morphology of the resulting HA), temperature, and the molar ratio of calcium to phosphate precursors. Synthetic HA typically exhibits a stoichiometric Ca/P atomic ratio of 1.67, which renders it less toxic and more stable compared to other CaPs. 

HA is widely used in scaffold designs and dental procedures [74]. In addition, it contributes to tooth enamel regeneration and tooth whitening when incorporated into toothpaste formulations [75]. In scaffold fabrication using HA, polymers serve as the matrix. Examples include combinations like PLA/HA, silk fibrin/HA, HA/TCP, and collagen/HA/PLA [76,77,78,79]. Examining the PLA/HA-based scaffolds depicted in Figure 2b, it becomes evident that their production is primarily motivated by considerations of bioactivity, processability, and mechanical properties. CaP, the main component of bone, finds extensive use in bone restoration applications. CaP exhibits a wide range of mechanical properties, with the Young’s modulus ranging from 70 to 120 GPa, flexural strength from 40 to 150 MPa, and compressive strength from 100 to 180 MPa. Furthermore, its modulus of elasticity falls within the range of 60 to 90 GPa. These characteristics indicate that CaP possesses mechanical properties conducive to bone tissue compatibility. However, their clinical application is limited by the relatively modest increase in the fracture and toughness properties [76], but when used in composite formulations, these shortcomings can be mitigated. In this respect, it is essential to highlight PLA as a synthetic polymer commonly used in tissue-engineering studies [80,81]. PLA, a thermoplastic polyester typically synthesized through microbial fermentation followed by separation and purification, is frequently preferred in studies that involve 3D-printing technology owing to several important characteristics. These include its biocompatibility, high mechanical strength, low cost, and compatibility with drug delivery systems. Blending with HA further broadens the medical application potential of this material [82,83,84]. Furthermore, it has been noted to contribute to the formation and proliferation of bone cells, particularly owing to its inorganic CaP content. However, a drawback lies in the challenges associated with extrusion due to the presence of HA. Nevertheless, it can be emphasized that the optimized PLA/HA composite provides bioactive, osteoinductive, and osteoconductive properties, thereby serving as a catalyst for further studies [76].

Another promising solution to address the identified shortcomings is the use of inorganic composites, such as synthetic wollastonite (CaSiO_3_), combined with HA [85]. CaSiO_3_ is biocompatible and promotes the growth of an apatite layer on its surface due to its high osteoconductivity and bio-resorption, which occur through the exchange of Ca^2+^ and SiO_3_^2−^ ions with the bio-organic environment [86,87]. These properties advance it as an excellent candidate for bone tissue replacement. Additionally, its porous structure and high mechanical strength support bone tissue integration and allow it to endure various mechanical loads [85]. The synthesis of such structures with adjustable morphologies and microstructures has been reported using sol-gel, hydrothermal, and precipitation technologies [88,89]. These methods are relatively straightforward and enable customization of the crystallite size and shape, as well as the surface curvature and roughness. To achieve specific bulk porosity by adjusting the pore size and shape, these techniques are often combined with template synthesis, utilizing colloidal organic and organo-inorganic templates [90].

Despite the numerous synthesis techniques available, including wet chemical precipitation, sol-gel processes, and hydrothermal methods, many face challenges related to economic viability and performance. Issues such as severe aggregation and agglomeration, wide particle size distribution, and phase impurities frequently occur, which complicate the production process and affect the quality of the final product. To address these issues, alternative approaches, such as extracting HA from natural sources, have been explored to produce high-quality HA more efficiently and cost-effectively. Thus, HA can be derived from both inorganic- and organic-based natural sources [91]. When synthesized from natural organic materials, HA often exhibits non-stoichiometric characteristics due to the limited ions present in its structure [92]. Biomaterials derived from both sources demonstrate excellent bioactivity and biocompatibility, although the processing costs may be more significant for inorganic materials [93]. Using natural sources such as eggshells and seashells offers the added benefit of incorporating Mg^2+^, Zn^2+^, and Al^3+^ cations, which improve the biological characteristics and foster bone regeneration, alongside ions such as F^−^, Cl^−^, and CO^3−^ [94,95].

Cleaning, boiling, demineralization, and re-mineralization are integral steps in the production of HA derived from bovine bones and fish wastes, respectively. HA obtained from bovine bones is particularly valued for its non-stoichiometric characteristics, rendering it suitable for bone transplantation [96]. The extraction of HA from natural resources like eggshells, seashells, and animal bones allows for the utilization of ions inherent in these materials for biological construction purposes. For example, eggshells, accounting for 11% of the total egg weight, comprise 94% calcium carbonate, 1% CaP, 4% organic components, and 1% magnesium carbonate. Parameters such as the grinding duration, calcination time, and temperature significantly influence HA production and dictate the properties of the final product [94]. This comprehensive approach highlights the significance of natural resources in HA production for a variety of biological applications.

It is noteworthy that HA derived from fish wastes (by-products of the fish industry) has been identified as a sustainable approach for production [97,98]. Apart from mitigating waste, this environmentally friendly approach aims to generate a versatile product appropriate for various applications, including bone regeneration, dental repair, and biomaterial manufacturing.

In recent research, HA derived from fish bone has been reported to be synthesized in the form of thin films using the pulsed laser deposition technique. Thus, in vitro experiments on osteoblasts, fibroblasts, and epithelial cells revealed that these thin films were not harmful, allowing them to be employed in various medical applications [72,97,98].

### 3.2. β-Tricalcium Phosphate

Next to HA, β-TCP is another ceramic material that shares the advantages of biocompatibility and biodegradation. It has the ability to promote the formation of new bone due to its osteoconductive properties. Unlike HA, β-TCP exhibits superior solubility, which makes it particularly effective for bone grafting in various dental and orthopedic applications [72,97,98]. It exists in both alpha and beta phases, with a Ca/P ratio of 1.5 [74]. β-TCP can be synthesized through methods such as solid-state reaction, thermal conversion, and precipitation [99]. However, the efficiency of β-TCP sintering is constrained by three factors: (i) the phase transition β → α-TCP, which occurs at 1115–1150 °C [100] and results in a volume increase [101], which typically leads to crack formation during the phase transition [102]; (ii) this transition occurs at a relatively low temperature, which hinders the achievement of high densities; and (iii) sintering is sub-optimal when pyrophosphate impurities are present (indicated by a Ca/P molar ratio of less than 1.50).

While they may have lower mechanical strength compared to HA, with a Young’s modulus ranging between 10 and 40 GPa, flexural strength between 20 and 50 MPa, compressive strength between 30 and 60 MPa, and modulus of elasticity between 5 and 15 GPa, they are still considered biocompatible and biologically active.

They can be used either in a mixture or separately. When combined, it has been demonstrated that the fragility and mechanical weakness of HA are eliminated. It has been observed that cell migration induced by the use of β-TCP is effective in terms of their incorporation and growth. Moreover, it was demonstrated that β-TCP is an ideal composite for the development of hard tissues [103], particularly for bone tissue engineering applications. In their study, Gmeiner et al. [104] synthesized two composites of PGA/β-TCP at ratios of 1:1 and 1:3 using solvent casting/leaching methods. The study indicated that the density of the PGA/β-TCP composite with a 1:3 ratio was higher than that of the PGA/β-TCP composite with a 1:1 ratio. In terms of in vivo studies, healing was observed to begin 30 days after the surgeries for both composites. After 90 days, it was indicated that the degradation rate of the PGA/β-TCP (1:3) composites was slow, with no significant damage to the bone observed. The study concluded that the mineralization values for the PGA/β-TCP composite with a ratio of 1:3 were superior [105]. Thus, it was indicated that these materials were more conducive to the growth of bone cells.

### 3.3. Bioactive Glasses

**Figure 4:**
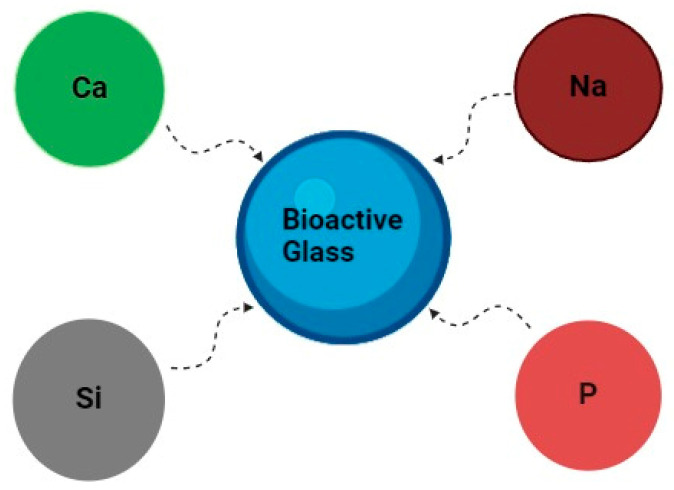
Elemental components of bioactive glasses.

BGs (Figure 4) are non-porous bioceramics found in a solid form. They are composed of silicon dioxide, calcium dioxide, sodium oxide, and phosphorus. BGs have gained attention due to their ability to bond with bone and stimulate osteogenesis [106]. They release ions that promote bone growth and possess antimicrobial properties. Composites combining BGs with polymers or ceramics aim to harness the benefits of each component, which further enhances the mechanical properties and biological performance [107].

When used in the ideal ratio (i.e., 50 wt.% SiO_2_, 25 wt.% CaO, and 25 wt.% Na_2_O) [48], they play a crucial role in increasing biocompatibility [108,109]. With a Young’s modulus ranging from 60 to 90 GPa, flexural strength in the range of 40 to 100 MPa, compressive strength between 60 and 120 MPa, and a modulus of elasticity between 30 and 50 GPa, BGs find application in various biomedical fields for bone tissue repair and regeneration. However, they have known fragility and insufficient mechanical strength [110,111]. Therefore, they present limitations, especially in load-bearing applications [74].

The density and mechanical properties, along with the fabrication potential, of BGs and silicate bioceramics have been assessed through various additive-manufacturing methods [112]. Both in vitro and in vivo studies have demonstrated their applicability in the human body [113]. However, limitations related to material processability and potential drawbacks during the production stages have also been identified [112].

A comparative overview of the main mechanical properties of HA, β-TCP, and BGs, which are commonly used in bone tissue scaffold construction, is presented in Table 1. The main mechanical properties, such as the Young’s modulus, flexural strength, compressive strength, and modulus of elasticity, are considered.

### 3.4. Calcium Silicates

CaSi bioceramics with high mechanical properties have become a research hotspot in the field of bone tissue repair biomaterials [6]. Bioactive CaSi may provide interesting advantages in relation to their chemistry as they expose silanol groups and release silicon [117]. CaSi demonstrated bio-interactive properties [118] and the ability to induce the differentiation of different populations of cells [119,120]. Moreover, CaSi can degrade and release Ca and Si ions, which can stimulate the osteogenic and angiogenic differentiation of cells [121].

The α-CaSi can be sintered at high temperatures without losing biological activity. In addition, α-CaSi can form an effective combination with the adjacent host bone in the body [122]. However, the main problem of α-CaSi is rapid degradation, which will lead to a significant increase in the pH of the microenvironment surrounding the scaffold. This might possibly cause cytotoxicity and could affect cell behavior [123,124]. Shuai et al. [125] constructed a more stable HA layer on the surface of CaSi scaffolds by hydrothermal treatment, which significantly reduced the degradation of CaSi scaffolds.

Natural polymers, including peptides (gelatin and collagen), natural poly-esters (polyhydroxyalkanoates, poly(-hydroxybutyrate and poly(-hydroxybutyrate-co-hydroxy valerate)) and polysaccharides (alginates, i.e., mannuronate/guluronate-based copolymers, cellulose, chitin, hyaluronic acid, pectin, and starch), have been used to prepare porous scaffolds for tissue engineering [126,127]. The inclusion of CaPs, such as dicalcium phosphate dihydrate (DCPD), in CaSi materials was demonstrated to enhance their biological properties and apatite-forming ability [128]. Moreover, when CaSi materials are used as filler in a polymeric matrix, such as PLA or PCL, the high alkalizing ability may counterbalance the acidic degradation products of synthetic poly-hydroxyl polymers [129]. These properties support their role as filler in tissue engineering.

To conclude, each bioceramic material has distinct advantages and limitations that dictate their suitability for specific applications. Thus, HA’s excellent biocompatibility and osteoconductivity make it ideal for applications that require long-term stability, such as dental implants and coatings for orthopedic implants. β-TCP’s biodegradability makes it more suitable for applications where temporary support and gradual replacement by natural bone are desired, such as bone grafts and scaffolds for bone defect repair. Finally, BGs and CaSi, with their bioactivity and ability to stimulate cellular responses, are particularly useful in applications where enhanced biological activity and integration with host tissue are critical, such as in composite scaffolds and bone defect fillers.

## 4. Methods Used for Scaffold Fabrication in Bone Tissue Engineering

The structures found in the human body exhibit a three-dimensional architecture. Consequently, structures intended to repair various parts of the human body must be designed and fabricated in perfect accordance with cell growth, extension directions, and other factors. In this respect, some important characteristics, along with the advantages and disadvantages of both CAD and conventional methods [130,131], such as thermal-induced phase separation, electrospinning, freeze-drying, gas foaming, particle leaching/solvent casting, fused deposition modeling, three-dimensional printing, stereolithography, and selective laser sintering, will be briefly introduced in the following sections and evaluated for their use in the biomedical field, specifically in bone tissue-engineering applications.

### 4.1. Thermal-Induced Phase Separation

**Figure 5:**
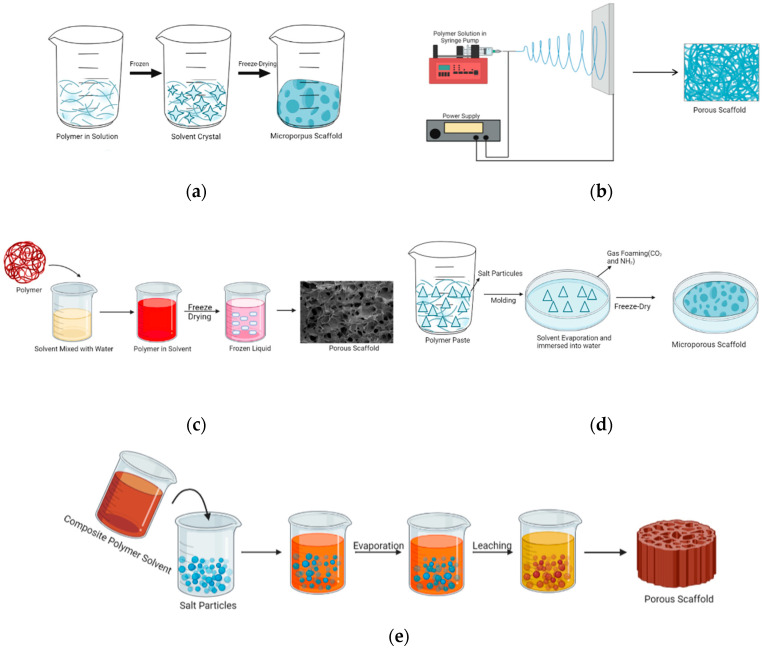
(**a**) Schematic of the thermal-induced phase separation method, (**b**) schematic of the electrospinning method, (**c**) schematic of the freeze-drying method, (**d**) schematic of the gas-foaming method, and (**e**) schematic of the particle-leaching/solvent-casting method.

Thermal-induced phase separation (TIPS) is a widely used method to produce porous materials with controlled architectures, especially in the field of biomaterials. Initially, a biocompatible polymer is dissolved in a solvent. This solution is then combined with a porogen, often ceramic particles. When subjected to temperature changes, phase separation occurs, which leads to the formation of different polymer- and solvent-rich phases. After solidification, typically achieved through gradual freezing, the solvent is removed. This process results in the formation of a porous scaffold [132]. Due to its ability to mimic natural extracellular matrices, scaffolds with controlled pore diameters are employed in various fields, including tissue engineering. Figure 5a illustrates the TIPS approach.

The TIPS technique was investigated by Szustakiewicz et al. [133]. In the study, they explored the characteristics of composite foams incorporating poly(L-lactide) (PLLA) and synthetic HA. This manufacturing method used both salt leaching (SL) and a newly developed TIPS technology. Various parameters, including the porosity, density, water contact angle, thermal stability, crystallinity, and compressive strength, were thoroughly evaluated for composites with varying concentrations of HA. The TIPS-SL approach, known for its precision, was used to meticulously prepare these composite materials. The findings of this study highlighted the beneficial relationship between improved mechanical characteristics, thermal stability, and cell proliferation capacity observed in composites with higher concentrations of HA. Moreover, it was unequivocally demonstrated that the TIPS-SL process provides a reliable mechanism for fabricating PLLA/HA composites with a highly stable porous structure. These advancements hold significant promise for the practical application of such composites in dynamic tissue-engineering applications, where the ability to mimic natural extracellular matrices is of paramount importance.

### 4.2. Electrospinning

The electrospinning technique, pioneered by John Francis Cooley and patented [80], represents a transformative process in which polymeric materials are converted into thin filamentous fibers under high pressure at elevated voltages (using an electrically charged needle) [134], typically ranging from 10 to 20 kV (see Figure 5b). Droplets of polymer solution form sprouts followed by the evaporation of solvent, which consequently leads to the formation of fine fibers that mat into a porous scaffold [131]. Widely acclaimed for its efficacy, this method is extensively employed in producing nonwoven nanofiber matrices. The electrospinning system comprises four integral components: (i) a syringe pump, (ii) a metallic needle, (iii) a high-voltage power supply, and (iv) a ground terminal, as depicted in Figure 5b [130,131,135].

Various polymeric materials, including PLGA, PCL, poly(ethylene oxide), polyvinyl alcohol (PVA), collagen, silk protein, and peptides, can be intricately associated with the electrospinning method.

A key advantage of this technique lies in the production of ultra-fine fibers with special orientation, characterized by the high surface area and high aspect ratio that control the pore geometry. All these characteristics make them well-suited for cell growth in both in vitro and in vivo applications [131]. Furthermore, these fibers exhibit notable mechanical strength, attributed to the homogeneous mixing of materials during the fiber formation process [130,131,136]. One should note here that the porous nature of a scaffold mainly influences the mechanical strength of the overall structure. Therefore, achieving an optimal balance between the porosity and the mechanical strength of the scaffold is one of the greatest challenges in tissue engineering [130]. Despite its simplicity and high efficiency, some limitations should be acknowledged, including the unsuitability of scaffold pore sizes for cell passage, the use of potentially toxic solvents, and the dependence of the process on numerous parameters, such as the applied voltage and solvent selection [130,131]. Cell seeding seems to be the main challenge of the electrospinning method. This issue could be overcome by using sacrificial biopolymers or cryospinning, which allow the creation of holes of the desired sizes in electrospun matrices [131].

### 4.3. Freeze-Drying

The freeze-drying method (Figure 5c), known as lyophilization, is based on freezing a polymer after dissolving it in a suitable solvent. After dissolving the polymer, the material is combined in one phase to form a heterogeneous mixture through emulsification. Following dissolution, the resulting polymer solution is cooled, which causes the solvent to evaporate through sublimation and results in the formation of a porous scaffold [135,137].

This method can be applied to several different polymers, including silk proteins, PEG, PLLA, and PLGA/poly(propylene fumarate) mixtures. It is advantageous to obtain high-dimensional pores. Another benefit is that the material does not require washing to remove the solvent [130,131].

With this method, which is also used to create porous structures, injectable gel structures with a sponge-like configuration can be achieved. Here, a polymer and a non-solvent are thoroughly sonicated, frozen in liquid nitrogen, and subsequently freeze-dried. Several advantages of scaffolds created with injectable gel can be mentioned here: (i) any shape of defect can be filled due to their good flow properties, (ii) the loading capacity for a range of cells and bioactive molecules can be achieved by simple mixing, (iii) a lack of residual solvents that may be present in preformed scaffolds, and (iv) no surgical procedures required for their placement [131,136].

Kordjamshidi et al. [136] investigated the microarchitecture of a bio-nanocomposite skeleton composed of naturally synthesized diopside and magnetite nanoparticles (MNPs). MNPs were tested with various weight fractions and produced using a freeze-drying process with sodium alginate. The mechanical and biological characteristics of CaSi ceramics were enhanced by incorporating a binary xCaO-ySiO_2_ base, including metal oxides. The hardness, elastic modulus, apatite production, biodegradation rate, wetting characteristics, roughness, and electrical conductivity were all assessed in porous bio-nanocomposite scaffolds. X-ray diffraction (XRD), transmission electron microscopy (TEM), and scanning electron microscopy (SEM) were utilized to investigate the composition, microstructure, and physical characteristics of the structures. The obtained results demonstrated that the addition of diopside bioceramics improved the mechanical and physical properties of the samples. Among the porous bio-nanocomposite skeletons, those with 10% weight MNPs exhibited superior performance as bone transplants for cancer therapy and hyperthermia. Furthermore, this scaffold proved to be a promising candidate for bone implantations and an efficient releaser of the medication celecoxib. The research also elucidated the connections between the MNPs concentration and various features, including the porosity, drug release kinetics, apatite formation, and biodegradation rate.

### 4.4. Gas Foaming

In this method (Figure 5d), inert gas-foaming agents such as effervescent salt (ammonium bicarbonate) are used. Carbon dioxide or nitrogen-inert gas-foaming agents are employed to create a porous structure within which the polymer is filled with gas bubbles [135,138].

Initially, a biodegradable polymer is melted by immersion in hot water and then mixed with salt particles to form a paste. As a result, the ammonium bicarbonate utilized decomposes into ammonia and carbon dioxide, which further initiates the formation of the porous structure within the polymer [139]. The porosity is created as gas escapes during the temperature increase or pressure reduction during polymerization [140]. This method can achieve a porosity level of 85–93% [139,140]. However, a potential disadvantage of this process is the cytotoxicity associated with the use of organic solvents. To mitigate this issue, minimizing the use of organic solvents is recommended. Rarely, the resulting scaffolds may exhibit a closed pore structure [139]. Additionally, this method offers the advantage of not requiring any filtration or washing procedure due to the absence of solvent usage. This helps prevent the loss of bioactive materials [109,130].

Kim et al. [140] developed a gas generation and particle dissolution (GF/PL) approach to fabricate composite scaffolds of PLGA/nano-HA without the use of organic solvents. The GF/PL technique enhanced the exposure of bioceramic nanoparticles on the scaffold’s surface. This resulted in linked porous structures without a skin layer and higher mechanical characteristics compared to SC/PL scaffolds. In vitro and when transplanted subcutaneously into athymic mice for eight weeks, both types of scaffolds seeded with rat skull osteoblasts demonstrated significant cell proliferation, alkaline phosphatase activity, and mineralization. Histological examination and calcium content measurements of the regeneration tissues at five- and eight-weeks post-implantation revealed greater bone production in the GF/PL scaffolds than in the SC/PL ones. These findings suggest that biodegradable polymer/bioceramic composite scaffolds created by the GF/PL approach are more effective for bone regeneration than those produced by the classic SC/PL method.

### 4.5. Particle Leaching/Solvent Casting

The particle-leaching/solvent-casting method (Figure 5e) uses a solvent combined with salt particles to dissolve a polymer [130,135,141]. After the salt particles mixed with the solvent are combined with the polymer, the solvent is evaporated, leaving behind the salt particles. Upon removal of these particles, the porosity of the polymer is achieved.

The scaffolds obtained by this method have a porous structure ranging from 50% to 90%. The possibility to adjust the porosity level of the fabricated structure represents an important advantage of this method. The preparation of a conducive environment for cell growth and development is thus facilitated [130,135]. Other advantages include the ability to fabricate structures with high porosity, model designs that can accommodate thin-walled membranes in 3D structures, and low processing costs. However, the drawbacks include long processing times due to the use of thin membranes and the use of potentially toxic solvents [135].

### 4.6. Fused Depositional Modeling

**Figure 6:**
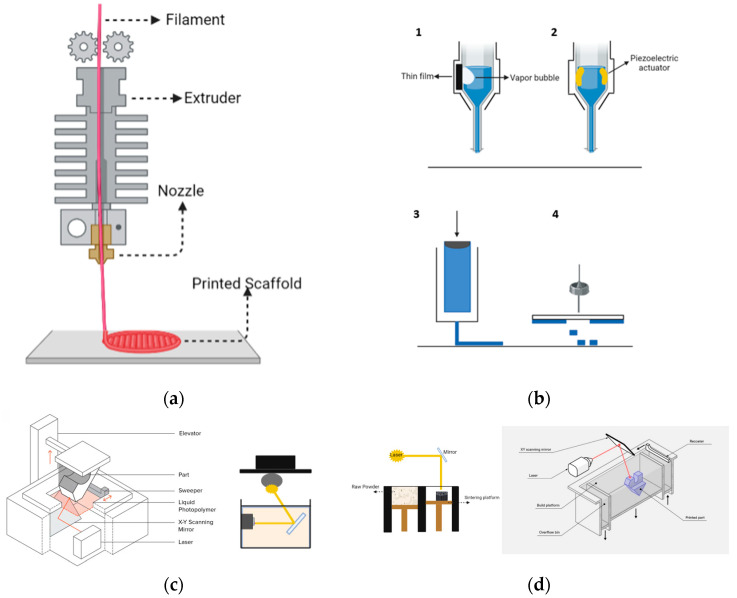
(**a**) Schematic of the fused deposition modeling method, (**b**) schematic of the three-dimensional printing method, (**c**) schematic of the stereolithography method, and (**d**) schematic of the selective laser-sintering method.

In fused depositional modeling (FDM) technique, the image source is generated from digital data obtained from imaging techniques such as computed tomography (CT), and magnetic resonance imaging (MRI). Initially, the geometric model of the present 3D image is designed. The geometric model is created using an extrusion head that moves in the X, Y, and Z directions (Figure 6a).

The extrusion head heats the thermoplastic polymer network inside, rendering it fluid. This allows the material to be deposited in thin layers. The 3D shape gradually forms as layers are added. The variety of polymers used for the extrusion method is generally more restricted compared to 3D modeling systems.

However, the parameters in the FDM technique have been tailored to process both simple (e.g., PCL) and composite (e.g., PCL/HA) materials. Moreover, it has been reported that this method can be used for both non-absorbable polymers and bioresorbable materials [142]. Janek and collaborators investigated the production and mechanical characteristics of composite filaments suitable for ceramic fusion-bonding technology. They created 1.75 mm diameter filaments using commercial HA powders and the thermoplastic polymer PVA. The mechanical strength and bending resistance of the filaments are influenced by their mechanical strength and fineness ratio. The composite filaments, comprising around 50% HA, were compared to a commercial filament composed of PLA and 27% gypsum. The tensile strength of the laboratory-prepared filaments is approximately three times that of the commercial filament. Employing the intrinsic Young’s modulus for measurement, the critical bending pressure computed using Euler bending analysis was 2.5–5.0 times lower than the highest filament compressive pressures recorded during bending simulation. The study aimed to develop a rapid and reliable approach to evaluate new formulations for the mechanical testing of laboratory-produced filaments.

FDM can print not only polymers but also metal and ceramic pieces. In such cases, the produced models need to undergo a sintering process to remove the binder polymer [113]. This technology offers several advantages, including the absence of solvents, the ability to adjust pore diameters, the production of pressure-resistant structures, strong pore connections, and low costs associated with both the machine and the filament material [130,131]. Two drawbacks that should be mentioned here are the irregular structures, which can be complex in solid modeling, and the resulting models with a low resolution [143].

### 4.7. Three-Dimensional Printing

An inkjet print head is utilized in 3D printing to deposit layers of material in droplets onto the platform. Each layer is applied as a fresh powder layer on top of the preceding one, gradually assembling a 3D object. Some advantages of this method include the use of low temperatures, absence of solvents, and ease of processing [113,143]. However, the disadvantages include the mechanical weakness caused by the material and difficulties in creating small details of the shape.

The first step in the process involves distributing fine powder on the powder bed. After a 3D model is created for printing, layer information is generated within the model program using the mesh algorithm. This layer information is then used to produce the material layers required for 3D printing.

The binder material is sprayed onto the existing powder layer using a technology similar to inkjet printing. This process is repeated for each layer until the aimed shape is complete. With this technique, the packing force created by the powder particles and the binding material affect the adhesive bond.

Composites used in 3D-printed scaffolds are particularly important for bone tissue engineering applications. With this technique, bioceramic powders, non-hydrogel-based polymers, as well as composites of these polymers, natural or synthetic hydrogels, are used as raw materials. Poly-β-hydroxybutyrate (PHB), PLGA, PVA, PLA, PCL, and polyurethanes are examples of some polymers that can be processed with this 3D method. They are prepared for 3D printing through melt-extrusion processes or solvent-based methods [144].

Tissue engineering is closely related to the 3D-printing method. Nowadays, 3D porous scaffolds are fabricated by blending biomaterials with cells. Cells harvested from humans are replicated and then transferred to the desired scaffold materials. The surface of the scaffold forms an extracellular matrix where cells proliferate, and structural and functional proteins are present. Cells seeded both inside and outside the scaffold are closely controlled.

The concept of additive manufacturing (AM) must be introduced here. This fabrication approach, which varies from standard manufacturing procedures, involves producing an entire object layer by layer. The AM technique is often utilized to develop biomimetic structures, which are designed in slices and manufactured using 3D technologies. Solids manufacturing is a rapid-prototyping technology often used in bioprinting applications to create solid objects by depositing material either layer by layer or slice by slice. This approach involves depositing solid layers or slices consecutively. It may be employed in various bioprinting applications, such as ink printing, mechanical working, micro extrusion, and laser forward transfer bioprinting (Figure 6b) [145,146,147].

### 4.8. Stereolithography

Stereolithography (SLA) is a laser-based 3D-printing method that creates 3D models, prototypes, and patterns layer by layer using photochemical processes. With this approach, a laser is utilized to crosslink light-sensitive polymers consisting of chemical monomers and oligomers. As the laser scans the surface of the liquid resin, the material solidifies. This results in the formation of each layer of the item, which is built up over time [148] (Figure 6c).

SLA is a 3D-printing process that constructs objects layer by layer, similar to FDM. In SLA’s top-down method, a transparent surface is placed near the liquid resin, and light is reflected onto it. The light cures the resin in a pattern, layer by layer, gradually building up the objects. Once a layer is cured, the structure is lifted to allow uncured resin to fill the space between the structure and the transparent surface. The process repeats for the next layer. After the item is fully produced, any remaining uncured resin is removed and the structure is exposed to UV radiation to create a more solid and stable assembly [149].

Compared to selective laser sintering, this method is cost-effective and can be used for large moldings [74]. Liu et al. [149] utilized stereolithographic 3D printing (SL-3DP) to fabricate HA scaffolds with varying pore sizes and investigated their mechanical and biological characteristics. HA scaffolds with pore sizes of 400, 500, and 600 μm were produced, with the 600 μm pore size exhibiting the highest compressive strength and biological stability. Examination of the macro- and microstructures of the scaffolds revealed their high potential for implant applications. Based on the cell proliferation and 3-(4,5-dimethyltiazol-2-yl)-2,5-diphenyltetrazolium bromide (MTT) assay findings, the H3 scaffold demonstrated the most promising prospects for bone defect healing. This study provided fundamental insights into the potential of SL-3DP-produced HA scaffolds in bone tissue engineering. Additionally, it can be employed to fabricate complex structures owing to its high accuracy, smooth surface quality, and rapid processing [149]. However, a drawback worth mentioning is the low mechanical strength of the fabricated products [150].

### 4.9. Selective Laser Sintering

Selective laser sintering (SLS) is a 3D-printing technology that uses a laser as its power source (Figure 6d). This method is versatile, as it can be applied to polymers, ceramics, or metals [135]. It is important to mention that SLS is sensitive to temperature variations. Therefore, precise temperature control is essential as it directly impacts the final product. The optimal temperature during the process depends on factors such as the glass transition temperature for polymers or the melting temperature for metals and ceramics. For instance, an uneven temperature distribution in biphasic tricalcium phosphate material used with SLS can result in wavy deformations in the desired structure [151]. 

Printing scaffold structures with the SLS method offers several advantages. One significant benefit is the capability to print using high-molecular-weight polyethylene. Additionally, the SLS settings can be adjusted to create intricate microstructures within the scaffold, which leads to high-resolution and customized designs [135,148]. Another advantage is the ability to fabricate scaffold structures in the desired form without requiring additional post-processing. Furthermore, scaffold structures produced using SLS exhibit high mechanical strength. Overall, SLS proves to be a highly efficient and effective method for scaffold fabrication [151].

The drawbacks of this technique include the high processing temperature required, the occurrence of post-processing phase transformation, and the necessity for injecting cleaning powder [135].

Although there are numerous fabrication methods available, their use is often restricted by their inherent disadvantages. In the subsequent sections, we will delve into the most recent advancements in the techniques employed for constructing scaffold structures, spanning from traditional to rapid manufacturing methods. Considering the rapid pace of technological development, it is foreseeable that 3D-printing technology will continue to expand in both scope and versatility, encompassing a wider array of materials and applications.

### 4.10. Current Challenges and Future Research Directions in the Development of Bioceramic Scaffolds for Bone Tissue Engineering

The development of bioceramic scaffolds for bone tissue engineering faces a multitude of complex challenges that somehow hinder their widespread clinical application. In the following paragraphs we will summarize these challenges with the aim of providing the reader with an overall image of the progress made in this direction.

- *Material properties*: One of the foremost issues is achieving an optimal balance between mechanical strength and biodegradability. In this respect, it is essential for bioceramics to be strong enough to support load-bearing applications but also to degrade at a rate that matches new bone formation.
- *Porosity and interconnectivity*: While high porosity is essential for bone in-growth and vascularization, it often compromises the structural integrity of the scaffold. Achieving a uniform pore distribution and interconnectivity without sacrificing strength is a challenging task.
- *Biocompatibility and bioactivity*: Ensuring that bioceramic scaffolds are fully biocompatible and do not induce any adverse immune responses are pivotal concerns. Additionally, enhancing the bioactivity of these materials to actively promote cell adhesion, proliferation, and differentiation remains a key focus.
- *Manufacturing techniques*: The development of scalable, cost-effective, and reproducible manufacturing techniques that can produce complex geometries and controlled pore architectures is still challenging. Each technique has its own limitations in terms of the precision, scalability, and the types of materials that can be used.
- *Clinical translation*: Finally, the transition from laboratory research to clinical practice is significant and involves overcoming regulatory barriers. In this respect, long-term stability and performance in vivo, along with clear clinical benefits, should therefore be demonstrated. It is important to stress that addressing these challenges requires a multidisciplinary approach and continuous innovation in materials science, engineering, and biomedical research.

As future research directions, one should emphasize the following:

- *Advanced manufacturing technologies*: continued development and refinement of additive-manufacturing techniques, such as 3D printing and robocasting, to achieve greater precision, complex architectures, and better control over porosity and interconnectivity.
- *Material innovations*: exploring new bioceramic composites and hybrid materials that combine the best properties of ceramics and polymers to enhance mechanical properties, bioactivity, and degradation rates.
- *Surface functionalization*: developing novel surface modification techniques to enhance the bioactivity and osteoinductive properties of bioceramic scaffolds to promote better cell attachment and proliferation.
- *Incorporation of various bioactive molecules*: embedding growth factors, peptides, and other bioactive molecules within the scaffold to stimulate bone regeneration and accelerate healing.
- *In vivo studies and clinical trials*: conducting comprehensive in vivo studies and clinical trials to evaluate the long-term performance, safety, and efficacy of bioceramic scaffolds in real-world applications.

#### SWOT Analysis

To provide the reader with a framework for understanding some of the strengths (**S**), weaknesses (**W**), opportunities (**O**), and threats (**T**) associated with the technologies introduced in Section 4, a SWOT analysis was performed. Guidance concerning future research and development efforts in the field of bioceramic scaffolds for bone tissue engineering was therefore introduced.

(i) Thermal-induced phase separation [132,152,153,154]:

**S**: Highly porous nanoscale structures; straightforward and low-cost process; precise control over the scaffold’s microstructure; adjustment of mechanical properties.

**W**: Use of solvents; small-scale manufacturing; limited material compatibility; thermal sensitivity; complexity in multiphase systems; quite complex and costly production process.

**O**: Innovative material combinations; tailored properties for patient-specific applications.

**T**: Technological competition; high costs of necessary raw materials or specialized equipment.

(ii) Electrospinning [134,152,155,156]:

**S**: High surface area to volume ratio; precise control of porosity; versatile and low-cost method; mimics natural extracellular matrix (enhanced cellular responses); scalability (mass production/commercial applications).

**W**: Poor mechanical strength (tendency of the threads to adhere together); solvent residues; slow production process; uniformity issues; low thickness structures.

**O**: Advancements in materials; tissue-engineering applications; drug delivery for localized therapy; customization (patient-specific designs).

**T**: Regulatory challenges; competition (emerging alternative fabrication techniques); higher costs; slow market acceptance (need for clinical trials).

(iii) Freeze-drying [137,152,157]:

**S**: Highly porous structures with low density and small pores (beneficial for cell infiltration and tissue in-growth); minimal use of harmful solvents.

**W**: Use of cytotoxic solvents; long process duration; scaffolds with low mechanical strength; high operational costs (due to high energy consumption).

**O**: Enhanced material combinations; incorporation of bioactive agents (i.e., drugs, growth factors) to enhance therapeutic potential; customization and personalization of scaffolds.

**T**: Technological competition and slow market acceptance of new scaffolding techniques; high costs associated with equipment and process.

(iv) Gas foaming [138,152,158]:

**S**: Easy to use technique; avoidance of toxic solvents use; scaffolds with sponge-like structure; high porosity and low density; controlled pore size (by adjusting gas concentration and foaming conditions); scalability (for industrial applications).

**W**: Heat developed during the compression molding process; isolated pores; continuous skin layer; poor mechanical strength; uniformity issues; residual gas entrapment within the scaffold (affects scaffolds’ structural integrity and functionality).

**O**: Innovative material blends; scaffold customization to meet specific patient needs.

**T**: High costs associated with the process.

(v) Particle leaching/solvent casting [141,152,159,160]:

**S**: High porosity (essential for cell infiltration, nutrient diffusion, and tissue integration); controllable pore diameter through salt particle size; versatility; cost-effective process.

**W**: Residual solvents; scaffolds with simple geometries; low mechanical integrity; uniformity and porosity issues; complex and time-consuming process.

**O**: Material innovations; scaffolds designed for personalized medicine.

**T**: Technological competition; economic factors (high costs associated with high-quality bioceramic materials and solvent removal processes).

(vi) Fused deposition modeling [152,161,162]:

**S**: Controlled porosity; solvent-free method; good mechanical properties; low-cost method; easily customizable for patient-specific needs (integration with digital design); efficient use of materials.

**W**: Limited choice of filament material; high heat requirements; inferior mechanical properties; medium accuracy; materials limitation range; surface finish (requirement for post-processing to achieve desired smoothness).

**O**: Multi-material printing (scaffolds with gradient properties or functionalized surfaces); incorporation of drug delivery mechanisms (for localized therapy and enhanced healing).

**T**: Technological advancements; high costs associated with bioceramic materials and process.

(vii) Three-dimensional printing [152]:

**S**: Fabrication of complex and highly precise scaffold geometries by integration with digital imaging (accurate replication of patient-specific anatomical structures); versatility in material use (flexibility in scaffold design); precise control over pore size, shape, and distribution.

**W**: Scaffolds with lower mechanical strength; post-processing requirements (i.e., sintering, surface finishing); high initial setup costs.

**O**: Material innovation; multi-material printing could create scaffolds with gradient properties or integrated functionalities (e.g., drug delivery systems).

**T**: Technological competition; high costs associated with technology and used materials.

(viii) Stereolithography [152,163]:

**S**: Fast process; high precision and fine resolution for the fabrication of complex scaffolds; smooth surface finish; material versatility; integration with digital design.

**W**: Support structure is required; use of toxic resins; brittleness and low mechanical strength of the scaffold; expensive equipment; material limitations; post-processing requirements (i.e., curing and washing, to remove residual resins).

**O**: Material development; multi-material printing; highly customized and patient-specific scaffolds (enhanced clinical outcomes and patient satisfaction).

**T**: Technological competition; high costs associated with technology and used materials.

(ix) Selective laser sintering [152,163,164,165]:

**S**: Control of shape architecture and porosity; support structure not required (reduced material waste and post-processing time); lack of solvents; scaffolds with good mechanical strength; integration with digital design.

**W**: Impossible to design sharp corners or complicated boundaries; high operating temperature; rough surface finish; difficulty in removal of residual powder; high energy consumption.

**O**: Incorporation of bioactive agents, drugs, or growth factors (enhancement of therapeutic potential); highly customized and patient-specific scaffolds.

**T**: Technological competition; high costs associated with technology and used materials.

## 5. Topology Optimization and Generative Design

The scaffold structure under design is characterized by its porous and intricate nature. Owing to this complexity, it features a multitude of pores, which contributes to a reduction in the overall weight. As a result, this reduction in weight translates to significant savings in both material consumption and production time. Moreover, the multi-porous nature of these structures closely mimics natural tissues, which offers ample space for cell adhesion and proliferation [166].

Generative design facilitates the precise design and production of the desired porosity numbers and sizes in a controlled manner. This is achieved through techniques such as topology or layout optimization, which calculate the optimal distribution of material within a structure, particularly under limited load conditions [166]. This process is also known as controlled layered bio-production.

Both generative design and topology optimization create ideal CAD drawings as outputs. However, unlike topology optimization, generative design imposes no restrictions based on product parameters such as the material and durability. It is capable of generating structures that may not be produced through other methods, particularly when combined with AM technique. The goal is to develop a process that yields multiple solutions [167].

The studies on generative design often aim to generate numerous minimal structures in 3D-compatible meshes and then assemble them. However, irregularly shaped structures hinder their full potential use. To address this issue, Voronoi diagrams are employed in generative design to create porous scaffolding structures [167,168,169]. Voronoi diagrams find application in various fields, including the study of artificial bone structures [167]. Artificial bones, such as bone scaffolds or implants, are engineered to replicate the structure and properties of natural bone tissue. Voronoi diagrams offer valuable insights into the geometric arrangement and material distribution within these artificial bone structures.

Recent articles [167,168,170] comprehensively assessed all the stages of scaffold structure production for bone tissue applications, including the evaluation of the appropriate porosity size, number, interpore connection, and trabecular thickness parameters. However, it is important to note that the suitability of scaffold structures for mimicking the trabecular structure of bone tissue has not yet been discussed [171].

**Figure 7:**
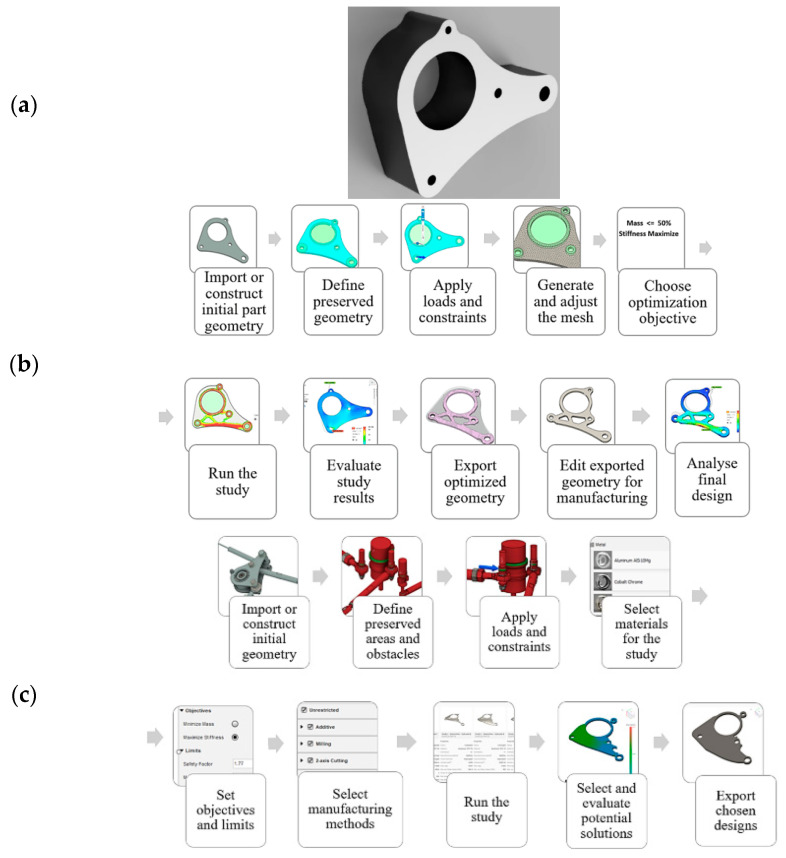
(**a**) Forces acting on the crank arm, (**b**) topology optimization workflow, and (**c**) generative design workflow. Reproduced with permission from Ref. [173].

In the next paragraph, a study that elucidates the difference between topology optimization and generative design using the example of a crank arm will be investigated [172]. The model in Figure 7a was meticulously crafted and encompassed considerations of the shape design, dimensions, and applied forces.

In this description, an example of the procedure employed in generative design is provided. The process has been adapted by creating separate files to facilitate shape optimization and generative design, utilizing the Autodesk Fusion 360 program. Specifically, the first file focuses on optimizing the shape of a single component, namely the crank arm. Additionally, the generative design encompasses all the necessary parts and links, which are included in the created STEP file. Optimizations have been applied without altering the initial weight and acting forces of the crank arm. The workflows available for topology optimization and generative design are shown in Figure 7b,c. The differences between the two techniques are highlighted [169,171].

The shape optimization module in Autodesk Fusion 360 Simulation conducted the topology optimization. Initially, a STEP model of the crank arm was imported into the simulation environment, outlining the outer limits of the design. A secondary material was selected, and the geometry at the connection points of the structure was defined. Load patterns were applied, and the prepared design underwent optimization according to one of the two objectives. One objective aimed to minimize the mass by effectively reducing the material used while maintaining strength. As a result, it achieved a reduction of up to 50% of the initially used material.

The material used remains unchanged and yields a single design suitable for topology optimization. However, the resulting design may not be ideal for direct production. Subsequently, after exporting the design, it must undergo editing to align it with production requirements [35,172].

When performing optimization with generative design, there is no constraint on the area. Instead of removing unnecessary material, the aim is to avoid zones termed obstacles by creating interconnected regions. In the initial model creation, the CAD model can either be built from scratch or utilized as an assembly of interconnected points.

After establishing the initial geometry, loads and constraints are applied to the model. Additionally, targets such as load-bearing capacities can be determined. Moreover, one can explore combinations not only with the specified material but also with different materials. For instance, the goal chosen here [173] was to minimize the mass while maintaining a safety factor. In generative design, both materials and manufacturing methods are selected, which leads to the advancement of numerous optimized designs [35,172].

## 6. Discussion

In this section, details related to some relevant studies reported in the literature are summarized in Table 2. Thus, the geometry of various scaffold structures, the materials and fabrication methods used, along with the porosity and mechanical strength values, are introduced and discussed in the following paragraphs. For more details, the reader is recommended to consult the appropriate references mentioned in the last column of Table 2.

**Figure 8:**
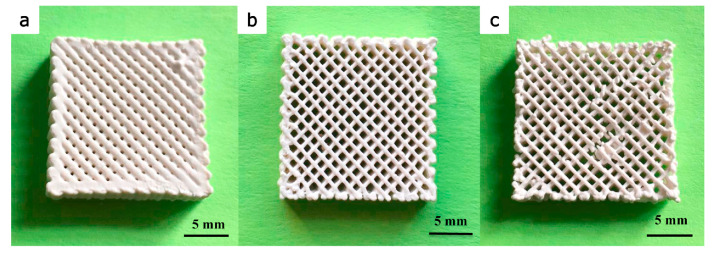
Green samples printed by 3DGP, with different printing speeds: (**a**) 3 mm/s, (**b**) 5 mm/s, and (**c**) 8 mm/s. Reproduced with permission from Ref. [174].

Shao et al. [174] fabricated three different 3D bio-scaffolds using the same material but at varying speed rates (Table 2, Figure 8).

It was observed that the most accurately shaped material was printed at an optimized speed of 5 mm/s, with the pore dimension of ~500 μm. The results indicated that the printed material degraded rapidly within less than two weeks, followed by a steady degradation thereafter. The weight loss of HA serves as a reliable indicator of the degradation properties in bone tissue engineering. The degradation rate maintained the mechanical strength and the degradation timeframe provided a sufficient duration for bone cell growth [174]. The study concluded that the nozzle diameter, polymer solution viscosity, and shear stress influenced the scaffold printing. Among the investigated printing methods for HA-containing polymers, 3D gel printing technology (3DGP) exhibited the highest efficiency, yielding a scaffold with 52% porosity and ~16.77 MPa strength.

In another study, Zeng et al. [184] demonstrated the capability of a specific 3D printing technology, known as digital light processing (DLP), to fabricate HA scaffolds with a square structure for bone tissue applications. To produce the HA scaffold, a photopolymer blend comprising various concentrations of HA powder and dimethyl sulfoxide (DMSO) was formulated, and through viscosity testing, a mass fraction of 30% HA was determined. Using these formulations, DLP technology was thus employed to fabricate HA scaffolds with a square pore architecture. As the HA concentration ranged from 0 to 30 wt.%, natural diffusion occurred within the groove, but the diffusion area decreased as the HA concentration increased. Consequently, a ceramic suspension with a mass fraction of 30 wt.% HA was chosen as the molding material for this experiment. A layer height of 0.05 mm and a printing speed of 20 mm/s were employed to fabricate the scaffold. The result was a square 3D model, which measured 21 × 21 × 3 mm^3^. Following the fabrication process, the sample underwent a drying phase at 85 °C for three hours to eliminate excess water from the HA scaffold. On one hand, the HA scaffold contained a photosensitive polymer that needed to be removed, while on the other hand, the mechanical properties of the scaffold needed improvement. Therefore, temperature-induced solidification sintering (TSS) was performed. TSS involves joining materials together by solidifying them at high temperatures, which results in material particles adhering to form a harder and denser structure. This method is commonly used for the strengthening and densification of materials. A compression model was then created using finite element analysis (FEA), and mechanical tests revealed that the specimens exhibited adequate compression performance. Subsequent in vitro cell culture experiments were conducted to assess the biological properties of the fabricated HA scaffold. Cell proliferation on the scaffold indicated its biocompatibility and suitability for cell growth and proliferation. The findings demonstrated that DLP technology can effectively construct ceramic scaffolds, and the presence of the photopolymer in the printed samples can be eliminated through high-temperature sintering. Consequently, ceramic parts with high compressive strength and biocompatibility can be produced.

**Figure 9:**
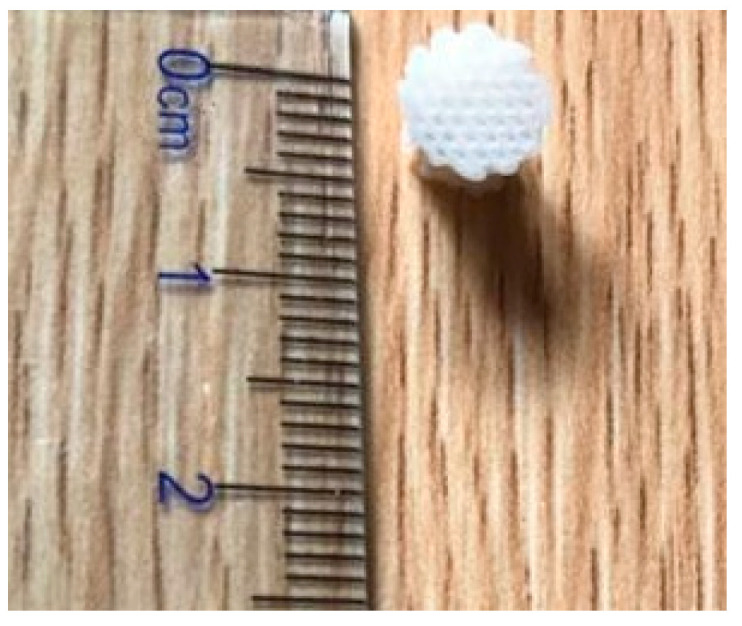
Polycaprolactone-hydroxyapatite scaffold in back view. Reproduced with permission from Ref. [175].

Liu et al. [175] reported on the production of 3D composite scaffolds composed of polycaprolactone and HA materials, loaded with heparan sulphate (HS) (Table 2, Figure 9).

In this study, a newly synthesized scaffold, denoted as high- and low-loaded HS, underwent in vitro and in vivo studies. The in vivo studies on rabbits were divided into four groups: (i) blank, (ii) control, (ii) low-concentration (50 μg/mL) HS, and (iv) high-concentration (500 ug/mL) HS. The in vitro studies showed that high-loaded HS had a significant inhibitory effect on osteoblast cell proliferation, while its impact in the in vivo studies was not substantial [35]. In comparison, the low-loaded HS scaffold demonstrated greater efficiency in healing bone defects. These findings suggest that HS exhibits excellent osteoinductive activity and offers a promising solution for bone regeneration. Consequently, these results might influence future studies. In this work, the scaffold model was constructed using Bioplotter CAD/CAM Rhinoceros software (v5.0 Educational) and printed via the layer-by-layer method. Its compression strength was measured using a static testing machine with a speed of 0.5 mm/min, which resulted in an inferred value of 5.18 MPa. Considering the material’s porosity at 70.8%, its strength can be readily correlated.

In another study, Chen et al. [176] reported on 3D-printed HA composite skeletons with superior mechanical characteristics. Due to its significant resemblance to bone minerals, HA is commonly utilized as a bone replacement material. The procedure involved combining HA nanoparticles, gelatine, and polymers. The study developed a method to produce composite skeletons from biodegradable materials such as HA, gelatine, chitosan (CHI), and carboxymethyl cellulose. Using the 3D-printing method, an ink comprised of HA and polymers was created, and this ink was used to produce HA composite scaffolds. The porous composite scaffolds were manufactured using a bioprinter with a compressed air extrusion cartridge. A circular column model was created for the bone defect model (D = 10 mm, H = 5 mm). Scanning electron microscopy (SEM) was used to study the morphology of the created scaffolds. The acquired images revealed the scaffolds’ microstructure and porosity. SEM micrographs of the combination of the varying ratios of CHI, gelatine, and HA revealed that the porosity of the fibers decreased with increasing concentrations of HA and CHI. A composition of 8.4% CHI weight and 60% HA resulted in more uniform and homogeneous structures compared to fibers prepared with 3.6% CHI weight and 40%, 50%, and 70% HA.

Thermal gravimetric analysis was used to characterize the thermal behavior of the composite skeletons. This investigation aimed to determine the thermal stability, degradation temperatures, and degradation quantity of the skeletons. Additionally, mechanical experiments were conducted to analyze the compressive strength and elastic characteristics of the skeletons. Compressive strength tests were performed to assess the period during which the skeletons could withstand maximum load. It was observed that the compressive modulus and strength of the skeletons increased with the HA and CHI levels. Meanwhile, the elastic characteristics were evaluated by analyzing the deformation rate and recovery capacity of the skeletons under compression. The test results indicate that the fabricated composite skeletons exhibited sufficient compressive and elastic properties. 

The findings revealed that 3D-printed HA composite scaffolds containing additional polymers exhibit superior mechanical properties compared to HA structures without additional composites. These scaffolds featured a porous structure compatible with bone tissue, thus promoting bone repair. 

**Figure 10:**
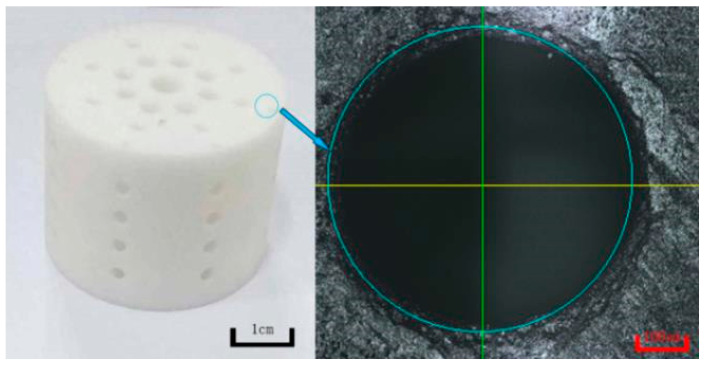
Hydroxyapatite scaffold fabricated by stereolithography. Magnification bars: 1 cm (**left image**), 100 nm (**right image**). Reproduced with permission from Ref. [177].

Wang et al. [177] utilized SLA to explore the design of HA scaffolds (Table 2, Figure 10).

HA was selected for its outstanding biological and mechanical characteristics. Initially, the femur bone was scanned using micro-CT, which yield 337 image slices. Subsequently, the CAD model of the femur bone was converted into a 2D layered STL file to facilitate SLA. In the subsequent step, commercial software package SolidWorks (v.2020) was employed to model the bone tissue on a computer before printing. An issue encountered in the study was the challenge of replicating irregular pore sizes using 3D printing technology. Consequently, eight scaffolds with varying sizes were fabricated, and their diameters were measured using a laser microscope. Subsequently, for in vitro studies, the scaffolds underwent sterilization by immersion in 90% alcohol for 10 h. After the sterilization process, bone marrow mesenchymal stem cells (BMSCs) were cultured. Upon reaching a certain confluency, they were seeded onto the scaffolds, and the structures were then placed in an incubator at 37 °C. Every two days, an MTT test was conducted to assess the cell proliferation.

**Figure 11:**
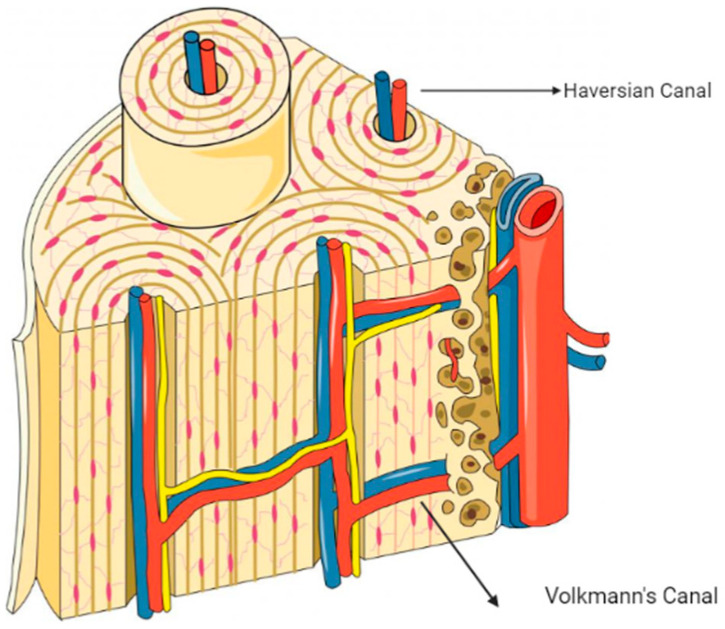
The representation of Volkmann’s and Haversian canals.

When investigating the mechanical properties of the fabricated scaffolds, it was observed that the increase in pore sizes had an impact on the compressive strength. This increase in the total porosity led to a decrease in the compressive strength from 9.2 to 2.8 MPa. Considering the natural bone structure, the compressive strength was also associated with the Volkmann’s and Haversian canals. Haversian canals, depicted in Figure 11, are straight, long, and run parallel to the femur, whilst Volkmann’s canals intersect with Haversian canals perpendicularly, extending randomly with numerous angles [185].

The canals house blood vessels that are essential for bone nourishment. In the fabricated scaffolds, perpendicular pores were designed to mimic Volkmann’s and Haversian canals. Interestingly, horizontal pores were found to negatively impact the scaffold’s compressive strength, whilst both vertical and horizontal pores had no significant effect on the seeded cell proliferation. Scaffolds fabricated via the SLA technique were further enhanced in terms of the mechanical and biological performance through sintering with HA. The observed weakness in the compressive strength, attributed to vertical pores resembling Volkmann’s canals, likely stemmed for their thinner nature compared to Haversian canals. This aspect affected the scaffold’s elasticity and strength despite consistent porosity levels. Ultimately, it was concluded that porosity positively influenced cell proliferation.

In another study, Baino et al. [178] used digital light processing stereolithography (DLP-SLA) technology to fabricate HA scaffolds. DLP-SLA is a 3D-printing process that uses UV light to polymerize materials layer by layer. The bone scaffold in this work was constructed using a light-curable slurry known as HA 480 E (Lithoz, Wien, Austria). This slurry comprises HA powder and a light-curable binder matrix containing solvent, reactive acrylate and methacrylate monomers, along with a photo-initiator. The CeraFab 7500 system was used to manufacture the scaffolds. A blue LED light source with a wavelength of 460 nm in the electromagnetic spectrum was utilized. The porosity and microstructure of the resulting HA scaffolds closely resembled those of natural bone tissue. While exhibiting a porous cylindrical shape with varying pore sizes and distributions, the scaffolds possessed a pore width of 580 μm, which was consistent with the standard reference range stated for trabecular bone. Permeability, a crucial factor with influence on oxygen, nutrient, and biological component transport, as well as tissue reactivity, was also assessed. The study demonstrated that the scaffolds exhibited acceptable permeability characteristics, which ensured adequate diffusion rates. Moreover, mechanical testing showed that the HA scaffolds exhibited high compressive strength and strong structural integrity, alongside high elastic modulus values. Consequently, it was concluded that DLP-SLA-fabricated HA scaffolds, which mimicked bone’s architectural, permeability, and mechanical features, hold promise for bone regeneration and tissue-engineering applications.

Tripathi et al. [179] explored the optimization of a 3D-printed gyroid bone scaffold through interactive modeling and experimental evaluation (Table 2). They focused on the trabecular bone scaffold, also known as cancellous bone tissue, which is typically found in the epiphyses of long bones (e.g., the femur). These porous tissues exhibit a sponge-like appearance [186]. In their study, the authors used a modeling framework to design a 3D gyroid scaffold, and they utilized professional software tools such as K3DSurf (v0.6.2), MeshLab (v1.0), and Netfabb (Ultimate v2017.2). K3DSurf facilitates 3D visualization of mathematical functions and curves. MeshLab serves as an open-source software for editing, cleaning, and analyzing 3D mesh data. Netfabb is a professional software for 3D modeling, preparation, correction, and printing. PCL was chosen as the material, and FDM technique was employed for scaffold fabrication. The gyroid shape was selected for its interconnected, smooth, and curved characteristics, which are conducive to vascularization—a crucial aspect in bone tissue engineering. The study notes the suitability of tetramethyl orthosilicate-based lattice structures for this purpose. The scaffold’s final porosity was set at 50% to achieve a balance between bone material and porosity, which enabled attachment or passage of seeded cells. The 3D scaffold was modeled using an implicit function-based equation as input.

**Figure 12:**
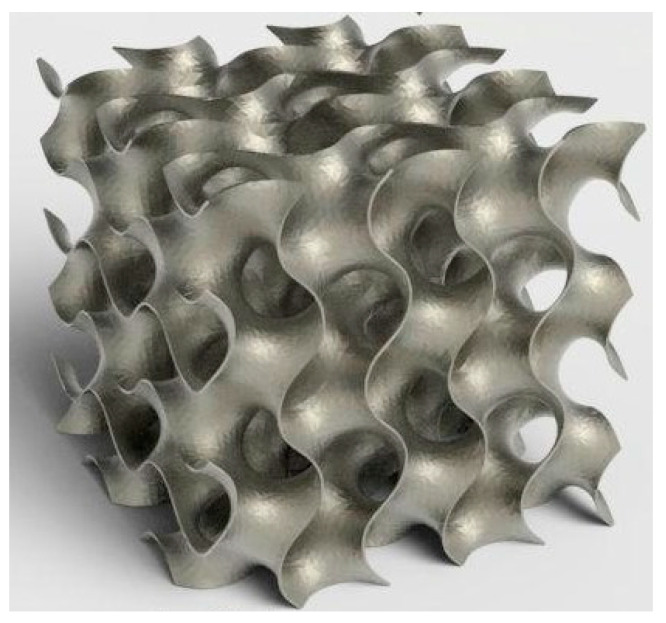
Representation of a 3D model corresponding to a gyroid scaffold [187].

The K3DSurf software was chosen for gyroid surface modeling due to its user-friendly interface and visualization capabilities. This software allows for grid resolutions of up to 100 × 100 × 100 pixels on each axis, where the grid resolution determines the smoothness of the 3D-printed model. Higher grid resolutions result in more detailed and smoother printed models. In this work, a 64 × 64 × 64 pixels grid resolution was selected. To ensure a well-defined boundary for unit cells during repetitive construction, a symmetrical domain was necessary. Consequently, the authors opted for a domain encompassing 64 (4 × 4 × 4) unit cells, as illustrated in Figure 12.

It is important to note that MeshLab software enables the rendering, editing, and conversion of meshes into various formats.

In another study, Alizadeh-Osgouei et al. [180] reported on the fabrication of gyroid skeletons by the FDM process using PLA biopolymer. The skeletons’ porosity and gyroid structure were optimized using design software. Gyroid skeletons with unit cell sizes of 2, 2.5, and 3 mm were created using Mathmod (v3.1) software and post-processed and scaled using Geomagic and Autodesk Netfabb. Compression and tensile tests were performed in two directions (structure direction and transverse direction) on dense PLA and porous PLA scaffold specimens with varying unit cell sizes. Furthermore, SEM analysis was used to investigate the skeleton morphology, and the proposed gyroid structure was validated. The mechanical properties of PLA gyroid scaffolds showed a result close to natural cancellous bone, which indicated that PLA gyroid scaffolds could enhance cell proliferation and create a favorable environment for tissue regeneration.

**Figure 13:**
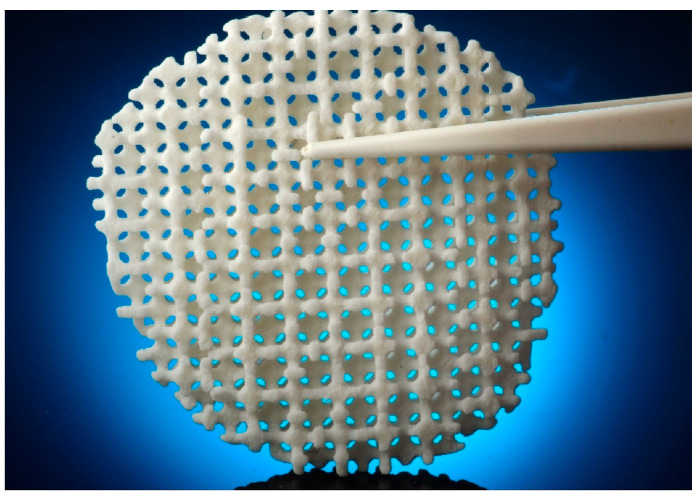
Patient-specific cranial implant demonstrator manufactured from the PLLA-1.0/CC (77/23) composite. The pore structures had a designed porosity of about 72% and a strut thickness of about 1 mm. Reproduced with permission from Ref. [181].

Gayer et al. [181] proposed a personalized design of the skull bone using biodegradable polymers (Table 2, Figure 13). 

The design had a diameter of 44 mm and a thickness of 11 mm, with a pore structure accounting for 72%, which was created using Autodesk Netfabb software (v2018.3 Ultimate). Initially, thermogravimetric analysis was employed to determine the most suitable polylactide/calcium carbonate ratio. A comparison was conducted by varying the operating power of the SLS method [181]. The article highlights the advantages of solvent-free manufacturing, and emphasizes the enhancement of the material chemical stability and reduction of the environmental impact. PLA and calcium carbonate were thoroughly mixed to form a composite material. A significant technical challenge in PLA processing via SLS is the potential occurrence of microporosity due to incomplete powder particle coalescence caused by high melt viscosity [188]. Studies from the literature report micropores as high as 46% or 55%. However, meticulous adjustment of the SLS process parameters enabled the production of test specimens with high strength (up to 75 MPa) and minimal microporosity (approximately 2%). Additionally, the SLS test specimens exhibited excellent cell compatibility with MG-63 osteoblast-like cells. 

In another study, Lee et al. [182] combined HA powder with camphene to create slurries with varying HA contents (10%, 15%, 20%, 25%, 40%, and 50%) through ball milling. These slurries were then poured into cylindrical molds and solidified at 42 °C. Thus, porous scaffolds with different HA concentrations resulted. It was observed that the scaffold architectures changed depending on the HA content, with the porosity and pore size decreasing as the HA concentration increased. The two-stage freeze-casting method effectively improved the connection between the inner and exterior sections of the scaffolds. As the porosity decreased, the compressive strength increased and offered a means to control the mechanical and structural qualities when constructing bone-like structures. The biocompatibility of the scaffolds was validated through in vitro cell attachment and proliferation assays. This demonstrated a significant enhancement in cell viability as pre-osteoblast cells spread across the scaffolds. Using successive freeze casting, inverted scaffolds with dense interiors and porous exteriors were produced. In this process, the slurry with a low HA content solidified in a smaller mold initially, followed by transfer to a larger mold, where the space was filled with slurries with a high HA content. Subsequently, the green stem underwent freeze-drying to sublimate camphor and induce pore formation, before being sintered at 1250 °C for two hours. The thicker component of these scaffolds provided load-bearing support while seamlessly integrating with surrounding tissue and bone.

**Figure 14:**
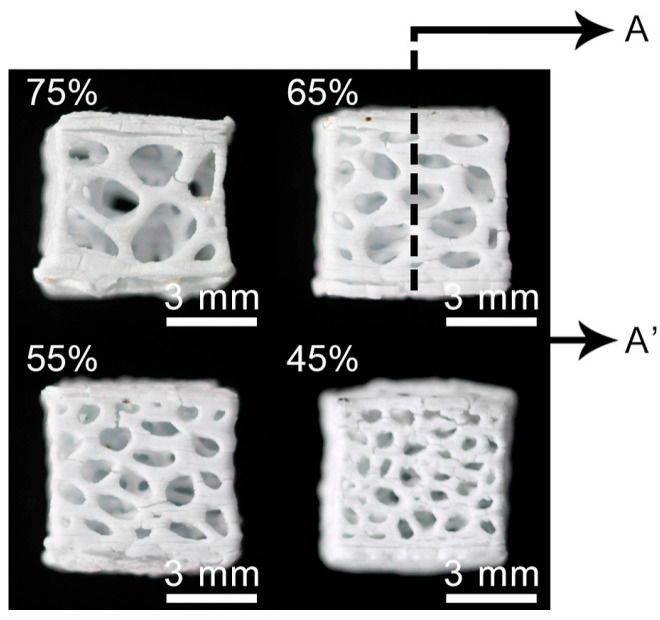
The 3D Voronoi based β-TCP porous scaffolds with decreasing porosity: 75%, 65%, 55% and 45%. Reproduced with permission from Ref. [183].

Liu et al. [183] reported on a Voronoi model (Table 2, Figure 14) designed to mimic trabecular bone. It featured irregular and interconnected pore structures, which are advantageous for tissue growth, including nutrient transport, cell proliferation, and vascularization.

The model incorporates both large and small pore sizes, which promote favorable osteoblast differentiation. Utilizing DLP as the 3D-printing technology, the study demonstrated the feasibility of achieving controllable porous structures. With DLP, the Voronoi model can be produced with high printing accuracy and offers a time-efficient and cost-effective fabrication process. Notably, the Voronoi mosaic pattern, based on Voronoi diagrams, is utilized on artificial bone implants to enhance compatibility with biological tissue. This surface-patterning approach facilitates increased cell attachment and tissue development [189], thereby promoting the implant’s success and the healing process.

Generative design, as previously mentioned, provides diverse solutions for structures with desired features and enables a multifaceted approach to achieve optimal outcomes. Generative design enables designers to explore various perspectives, which results in a wider array of feasible solutions. This facilitates a more efficient and effective design process and aids in the selection of the optimal design alternative. Essentially, generative design fosters creativity, innovation, and enhances the design process. Using this approach, scaffold structures can be fabricated using suitable methods by modeling them in different sizes. Studies have demonstrated that such an approach yields favorable mechanical performance and cytocompatibility for orthopedic implants [172]. Moreover, 3D Voronoi porous scaffolds with controllable porosity and pore size were designed with the CAD software Rhinoceros 6 and the Grasshopper plug-in (v0.9.0076), and they were suitable for finite element analysis. β-TCP porous ceramic pieces designed with 3D Voronoi structure were fabricated and showed a high structural similarity with natural trabecular bone. The compressive strength of the 3D Voronoi model trabecular-like β-TCP scaffolds was generally found to be between 0.8 and 4.1 MPa, with a porosity of 45–75%, and a pore size of 360–1200 μm. It was also observed that the compressive strength values increased as the pore size decreased [183].

### 6.1. Requirements for a Scaffold to Pass Clinical Trials

The journey of a scaffold from the laboratory to clinical application involves rigorous testing to ensure its safety, efficacy, and functionality. In the following paragraphs, some of the key types of assays, along with some considerations for scaffold fabrication technologies, will be briefly introduced with the aim of providing the reader with an overall image of the requirements of clinical trials that could generate effective solutions for future tissue-engineering applications.

#### 6.1.1. Biocompatibility Assays

Biocompatibility is paramount for any scaffold intended for clinical use. These assays evaluate whether the scaffold materials elicit any adverse biological reactions. The main tests include the following:

- *Cytotoxicity tests*: Assess whether the scaffold materials are toxic to cells in vitro. Common assays include MTT reagent, (2,3-bis-(2-methoxy-4-nitro-5-sulfophenyl)-2H-tetrazolium-5-carboxanilide)—XTT reagent, or live/dead staining methods to evaluate cell viability and proliferation.
- *Hemocompatibility tests*: Essential for scaffolds interacting with blood, these tests determine if the scaffold causes hemolysis or other adverse reactions in blood components.
- *Sensitization and irritation tests*: Determine if the scaffold causes allergic reactions or irritation in tissues. These tests are usually conducted using animal models.

#### 6.1.2. Mechanical Tests

Scaffolds must possess appropriate mechanical properties to support tissue regeneration and function. The key tests include the following:

- *Tensile and compressive strength tests*: Measure the scaffold’s ability to withstand forces without deforming or breaking. These tests are crucial for scaffolds used in load-bearing applications.
- *Elastic modulus and flexural tests*: Assess the stiffness and flexibility of the scaffold and ensure that it can mimic the mechanical properties of the target tissue.
- *Durability tests*: The scaffold should maintain its structural integrity over the desired period of implantation.

#### 6.1.3. Porosity and Interconnectivity Tests

These assays include the following:

- *Porosity tests*: The scaffold must have an optimal pore size and porosity to facilitate cell infiltration, nutrient flow, and waste removal.
- *Interconnectivity tests*: Pores should be interconnected to allow for vascularization and tissue in-growth.

#### 6.1.4. Biological Activity Assays

These tests ensure the scaffolds support cell attachment, proliferation, differentiation, and tissue formation:

- *Cell attachment and proliferation assays*: Often involve seeding cells onto the scaffold and using assays like DNA quantification, Alamar Blue, or WST-1 reagent to measure cell growth.
- *Differentiation assays*: Evaluate if the scaffold can promote stem cell differentiation into the desired tissue type. These assays might include measuring specific markers using techniques like real-time polymerase chain reaction, Western blotting, or immunocytochemistry.

#### 6.1.5. Scaffold Fabrication Technologies

When developing scaffolds, the selection of the appropriate fabrication technology is crucial to achieve optimal results in laboratory testing. Each technology has its own advantages and must be chosen based on the specific requirements of the intended application.

- *Material selection*: Materials should be chosen based on their biocompatibility, biodegradability, and bioactivity characteristics. For example, scaffolds made from materials like PLA and PGA provide mechanical support while being biodegradable. The incorporation of bioactive materials like CaSi or DCPD can enhance the scaffold’s ability to promote bone regeneration by providing essential ions for mineralization.
- *Fabrication techniques*: Different fabrication techniques offer various advantages.
- *Incorporation of bioactive molecules*: Scaffolds’ overall performance can be enhanced through the incorporation of bioactive molecules (i.e., growth factors, drugs) to promote cell differentiation and tissue regeneration. Stem cells can also be integrated within the scaffold to provide a source of regenerative cells.

#### 6.1.6. Sterilization Assays

These tests must demonstrate that the scaffold can withstand sterilization processes without losing its properties.

#### 6.1.7. Manufacturing and Quality Control Tests

These tests include the following:

- *Reproducibility tests*: The manufacturing process should consistently produce scaffolds with uniform properties.
- *Scalability tests*: The process should be scalable for mass production while maintaining quality.
- *Regulatory compliance tests*: Adherence to Good Manufacturing Practices (GMPs) and other regulatory standards.

#### 6.1.8. Preclinical Tests

These assays evaluate how the scaffold degrades over time in the living body. It is critical for the scaffold to degrade at a rate that matches tissue regeneration.

- *In vitro degradation tests*: Involve immersing the scaffold in a simulated body fluid and measuring the weight loss, structural integrity, and changes in mechanical properties over time.
- *In vivo degradation studies*: Conducted in appropriate animal models to observe the degradation behavior in a living system; they provide a more realistic assessment.

#### 6.1.9. Clinical Trials

These assays include the following:

- *Phase I*: Assess safety and preliminary efficacy in a small group of patients.
- *Phase II*: Evaluate efficacy and side effects in a larger patient group.
- *Phase III*: Confirm efficacy, monitor side effects, and compare with standard treatments in a larger population.
- *Phase IV*: Post-market surveillance to monitor long-term effects and performance.

#### 6.1.10. Regulatory Approval

This procedure includes the following:

- *Documentation*: Comprehensive documentation of all the testing, including preclinical and clinical data, manufacturing processes, and quality controls.
- *Submission*: Submission of a regulatory dossier to relevant authorities (e.g., Food and Drug Administration, European Medicines Agency) for review and approval.
- *Approval*: Obtaining regulatory approval based on the safety and efficacy data.

## 7. Conclusions and Future Perspectives

This work presents an overview of the key techniques employed in scaffold fabrication for bone tissue engineering. It has therefore shown that the development of bioceramic scaffolds (fabricated from hydroxyapatite, beta-tricalcium phosphate, bioglasses and calcium silicates) for bone tissue engineering presents a promising avenue for addressing critical clinical needs in orthopedic and dental applications. Current fabrication techniques, including thermal-induced phase separation, electrospinning, freeze-drying, gas foaming, particle leaching/solvent casting, fused deposition modeling, three-dimensional printing, stereolithography, and selective laser sintering, were indicated to offer unique strengths and face specific issues. As there is currently no established technique for artificial bone repair, ongoing research aims to develop the ideal scaffold structure. 

Among the current challenges in bioceramic scaffold fabrication, one could consider those related to mechanical strength, biocompatibility, and bioactivity, porosity, pore size, and interconnectivity, scalability and costs, along with regulatory and market acceptance. The ultimate goal is to produce a scaffold structure that closely mimics natural bone. Various scaffold shapes/geometries are discussed in this work, including square, cylinder, pored-cylinder, gyroid, circle, and Voronoi. The reason for the continuous exploration of ideal scaffolds is that structures formed by combining small geometrical shapes often fail to meet the requirements of natural bone porosity. Thus, another important challenge is to achieve precise control over the desired scaffold structure. Consequently, the employed fabrication methods are constantly evolving to produce structures that closely resemble natural bones.

In this respect, rapid prototyping methods have been adopted. Essentially, using the layer-by-layer fabrication technology, scaffold structures with desired properties and porosity can be designed. Thus, challenges encountered during production, as well as the determination of optimal results during the design phase through the exploration of alternative solutions, have been addressed. Generative design, developed to overcome the limitations of topology optimization previously used in this field, has proven successful. With generative design, multiple solutions are provided to achieve the desired structural features, which further facilitate the attainment of the intended outcome. Hence, one can note that through productive design practices, scaffold structures can be easily fabricated using suitable production methods. Viable solutions to model and design them in a variety of geometries and sizes are therefore offered.

Last but not least, while significant progress has been made in the development of bioceramic scaffolds for bone tissue engineering, addressing the current challenges requires a multifaceted approach. This involves advanced material science, innovative fabrication techniques, and thorough clinical validation. Future research on bioceramic scaffolds’ fabrication should therefore aim to enhance their mechanical properties, biocompatibility, and functionality, while also making them more cost-effective and scalable for widespread clinical applications.

## Figures and Tables

**Table 1:** The values of the Young’s modulus, bending and compressive strength, and modulus of elasticity corresponding to hydroxyapatite (HA), β-tricalcium phosphate (β-TCP), and bioactive glasses (BGs).

Bioceramic Material	Young’s Modulus [GPa]	Bending Strength [MPa]	Compressive Strength [MPa]	Modulus of Elasticity [GPa]	Ref.
HA	70–120	40–150	100–180	60–90	[114]
β-TCP	10–40	20–50	30–60	5–15	[115]
BGs	60–90	40–100	60–120	30–50	[116]

**Table 2:** Different shapes/geometries, obtained porosity and mechanical strength of bio-scaffold structures fabricated from various materials by 3D-printing techniques.

Shape/ Geometry	Material	Fabrication Technique	Obtained Porosity [%]	Mechanical Strength [MPa]	Ref.
Square	Acrylamide monomer, HA powder	3D Gel Technology	52.26	16.77	[174]
Cylinder	PCL(poly-caprolactone)- HA, Heparan Sulfate	3D Printing Technology	70.8	–	[175,176]
Pored-Cylinder	HA-Resin	SLA-Stereolithography	49.3–72.6	5.6–18.4	[177,178]
Gyroid	PLA Filament	FDM-Fused Deposition Modeling	49–50	7.32–8.53	[179,180]
Circle	Polylactide/Calcium Carbonate	SLS Method-Selective Laser Sintering	72	–	[181,182]
Voronoi	m β-TCP-Po	SLA-Stereolithography	45–75	0.8–4.1	[183]
